# The queen conch mitogenome: intra- and interspecific mitogenomic variability in Strombidae and phylogenetic considerations within the Hypsogastropoda

**DOI:** 10.1038/s41598-021-91224-0

**Published:** 2021-06-07

**Authors:** Salima Machkour-M’Rabet, Margaret M. Hanes, Josué Jacob Martínez-Noguez, Jorge Cruz-Medina, Francisco J. García-De León

**Affiliations:** 1 Laboratorio de Ecología Molecular y Conservación, Departamento de Conservación de la Biodiversidad, El Colegio de la Frontera Sur (ECOSUR), Avenida Centenario Km 5.5, C.P. 77014 Chetumal, Quintana Roo Mexico; 2 Department of Biology, Eastern Michigan University, Ypsilanti, MI USA; 3 Laboratorio de Genética para la Conservación, Centro de Investigaciones Biológicas del Noroeste (CIBNOR), S.C. Ave. Instituto Politécnico Nacional No. 195, Col. Playa Palo de Santa Rita, AP 128, 23096 La Paz, Baja California Sur Mexico; 4 Comisión Nacional para el Conocimiento y Uso de la Biodiversidad (CONABIO), Av. Liga Periférico - Insurgentes Sur, No. 4903, Col. Parques del Pedregal, Alcaldía Tlalpan, 14010 Ciudad de México, Mexico

**Keywords:** Mitochondrial genome, Genome evolution, Phylogenetics

## Abstract

*Aliger gigas* is an economically important and vulnerable marine species. We present a new mitogenome of *A. gigas* from the Mexican Caribbean and use the eight publicly available Strombidae mitogenomes to analyze intra- and interspecific variation. We present the most complete phylogenomic understanding of Hypsogastropoda to date (17 superfamilies, 39 families, 85 genera, 109 species) to revisit the phylogenetic position of the Stromboidea and evaluate divergence times throughout the phylogeny. The *A. gigas* mitogenome comprises 15,460 bp including 13 PCGs, 22 tRNAs, and two rRNAs. Nucleotide diversity suggested divergence between the Mexican and Colombian lineages of *A. gigas*. Interspecific divergence showed high differentiation among Strombidae species and demonstrated a close relationship between *A. gigas* and *Strombus pugilis*, between *Lambis lambis* and *Harpago chiragra*, and among *Tridentarius dentatus*/*Laevistrombus canarium*/*Ministrombus variabilis*. At the intraspecific level, the gene showing the highest differentiation is ATP8 and the lowest is NAD4L, whereas at the interspecific level the NAD genes show the highest variation and the COX genes the lowest. Phylogenomic analyses confirm that Stromboidea belongs in the non-Latrogastropoda clade and includes Xenophoridea. The phylogenomic position of other superfamilies, including those of previously uncertain affiliation, is also discussed. Finally, our data indicated that Stromboidea diverged into two principal clades in the early Cretaceous while Strombidae diversified in the Paleocene, and lineage diversification within *A. gigas* took place in the Pleistocene.

## Introduction

The Queen conch, *Aliger gigas* Linnaeus 1758 (Gastropoda, Strombidae; previously *Strombus gigas* Linnaeus, 1758; synonym *Lobatus gigas* [Linnaeus, 1758]), is restricted to coastal regions of the western Atlantic from Bermuda and southern Florida to Brazil^1^ from 5–20 m in depth^2^. *Aliger gigas* is one of the largest gastropods in the world with a siphonal length up to 30 cm^3^, and has high ecological, esthetic, and economic value^4^. The economic value of *A. gigas* resides principally in the commercial and nutritional value of its meat^5^. This mollusk is considered as the second most important fishery resource in the Caribbean after the spiny lobster (*Panulirus argus* Latreille, 1804 ^4,5^). Due to overfishing and poaching, the species is listed as a vulnerable commercial species (Appendix II, CITES, 1992; in^1^). Populations continue to be decimated due to overexploitation and habitat loss^6^. Many countries have implemented management strategies at the regional level, though an international synchronization of management and conservation practices is also in play to try to recover stock populations^1,6^. *A. gigas* has been extensively studied to better understand its biology e.g.,^4,7^, ecology e.g.,^8,9^, population genetic structure e.g.,^1,10^, conservation status e.g.,^6,11^, and phylogenomic position^12–15^.

The SPF Stromboidea (Rafinesque, 1815) belongs to the Hypsogastropoda clade within the higher taxonomic group Caenogastropoda^15,16^. The membership, taxonomy, and relationships within Hypsogastropoda, however, have been in considerable flux in the last two decades. Bouchet and Rocroi^17^ considered the Hypsogastropoda clade to be comprised of two groups: the Littorinimorpha (including Strombidae) and the Neogastropoda (Supplementary Table S1). More recently, Bouchet et al.^18^ reorganized the clade Hypsogastropoda. This revision also comprises two major groups, the superorder Latrogastropoda is home to the Neogastropoda and six SPFs from the Littorinimorpha as “taxa of uncertain position” (Calyptraeoidea, Cypraeoidea, Ficoidea, Tonnoidea, Xenophoroidea, and of particular interest, Stromboidea), and the Non-Latrogastropoda^13^ which contain other Littorinimorpha SPFs. Genetic and genomic studies proposed Tonnoidea SPF as an early branching lineage within the Neogastropoda e.g.,^12,13,16^ supporting the Bouchet et al.^18^ classification. The phylogenetic position of Xenophoridae has been debated as a sister clade to Stromboidea^16,19^ or embedded within Stromboidea^15^.

Mitochondrial genomes have become popular in elucidating gastropod taxonomic controversies e.g.,^20,21^ and have proven particularly useful in the resolution of uncertainties in the Caenogastropoda e.g.,^12,13,22^. Ascertaining the phylogenetic position of family Strombidae has been particularly difficult; studies have considered them as belonging to Littorinimorpha^14–16,23^ or to Latrogastropoda e.g.,^13,21,22^. Identifying the closest relatives to Strombidae has also been challenging, and has been closely associated with Xenorphoridae^15,16,19^, Cypraeoidea^12^, Tonnoidea^14,23^, or sister to the Latrogastropoda or even Neogastropoda e.g.,^13,21,22^. The recent proliferation of available mitogenomes provides an opportunity to conduct detailed analyses to better understand and confirm the phylogenetic position of Strombidae within the Hypsogastropoda clade.

Paleontological studies suggest the Stromboidea originated within the Cretaceous (and also Triassic or Jurassic)^16,24–26^. Fossils suggest that Strombidae probably diverged from Aporrhaidae in the late Cretaceous, initially with very low diversity followed by a rapid taxonomic radiation in the early Cenozoic^27^. Strombidae became the most species-rich family of Stromboidea during the Cenozoic as it expanded its geographic range^27^. Many fossils are reported from the Eocene to Pliocene e.g.,^24,28–30^ with a possible radiation at mid-latitude areas in the early Eocene^27^.

Historically, population genetic studies have been based on a limited number of markers, for example microsatellites e.g.,^10,31^, which allow for interpretation of intraspecific population structure^32^. Also, some mitochondrial genes (e.g., COX1) have been extensively used for intra- or interspecific comparisons e.g.,^33,34^. Recently, the use of mitogenomes in investigations of intra- and interspecific variation has become the best tool available e.g.,^14,32,35^. It is useful to identify which of the 13 PCGs of the mitogenome have adequate polymorphisms to resolve population genetic and/or phylogenetic questions^36^. Within family Strombidae only one study^14^ has used mitogenomes to evaluate interspecific relationships (in *Harpago* and *Lambis*), and until now, no research has employed the complete mitochondrial genome to evaluate lineage diversity within Strombidae. Given the vulnerable conservation status of *A. gigas*, it is important to assess intraspecific variation and population divergence within the species to guide future management decisions. Our work can further identify polymorphic mitochondrial genes for focused population level studies.

We take advantage of the large number of mitogenomes in clade Hypsogastropoda available on GenBank, including the very recent Stromboidea mitogenomes published^15,37^, and the two mitogenomes of *A. gigas* to: (i) present and describe a completely annotated mitogenome of *A. gigas* from the Mexican Caribbean, (ii) quantify intraspecific variation between our newly generated *A. gigas* mitogenome with that of one from off the coast of Colombia, (iii) evaluate interspecific variation among eight Strombidae species, (iv) confirm the phylogenetic position of Stromboidea and its relationship with Xenophoridae, as well as the relationships between eight Strombidae species using 110 mitogenomes, and finally, (v) estimate divergence times throughout Hypsogastropoda, Stromboidea, Strombidae and *Aliger*, respectively.

## Results and discussion

### Mitogenome: structure and organization

The complete mitochondrial genome of *Aliger gigas* from the Mexican Caribbean was sequenced, assembled, and deposited in GenBank (accession number MZ157283). The total length of the mitogenome is 15,460 bp which is consistent with other mitogenomes obtained from Strombidae species: *Aliger gigas* 15,461 bp^11^, *Lambis lambis* 15,481 bp^14^, *Harpago chiragra* 15,460 bp^14^, *Tridentarius dentatus* 15,500 bp^15^, and *Laevistrombus canarium* 15,626 bp^37^. *Conomurex luhuanus*^38^ and *Strombus pugilis*^15^ have longer total lengths, 15,799 bp and 15,809 bp, respectively, due to the presence of a large, non-coding region (428 bp and 436 bp respectively) between the tRNA-Phe and the COX3 genes proposed as a candidate for D-loop^38^. No D-loop has been annotated in other Strombidae species. *Ministrombus variabilis* presented a shorter mitogenome with 15,292 bp due to the lack of tRNA identification (only 18 resolved)^15^. Considering all other mitogenomes of Hypsogastropoda included in this study, only ten species (belonging to three families Conidae, Littorinidae, Cypraeidae) show annotations for D-loop.

**Figure 1:**
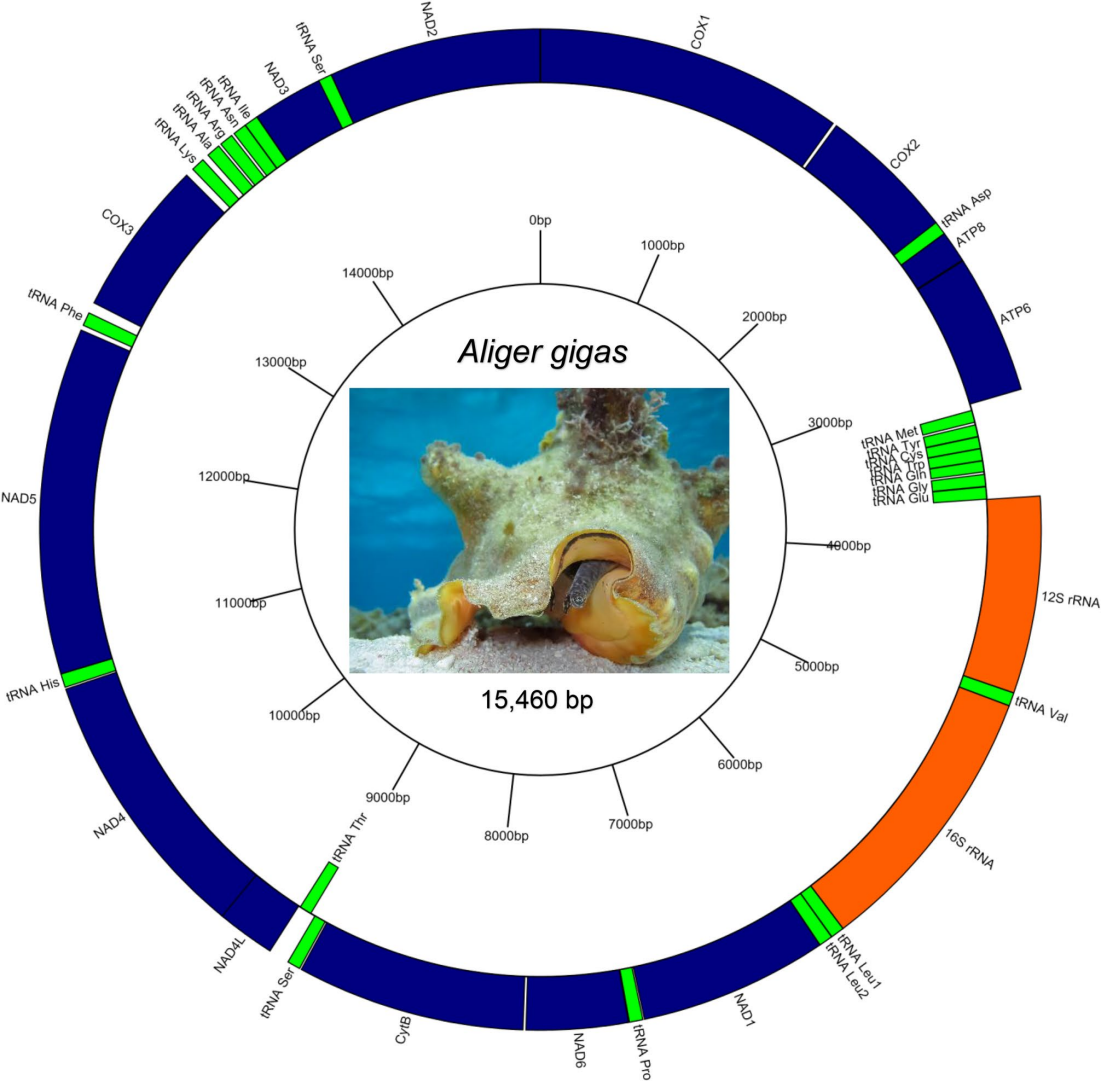
Mitochondrial genome map of *Aliger gigas* (GenBank MZ157283). All 37 genes are represented outside of the circle (direction 5’ → 3’) and to the inside (direction 3’ → 5’) in order and relative size and including non-coding regions. Protein coding genes (blue), transfer RNAs (green) are identified using the three letters corresponding to their amino acid, and ribosomal RNAs (orange) are presented. Photo by: HBahena/ECOSUR.

The *A. gigas* mitogenome presented here contained 13 PCGs, two rRNAs (12S and 16S), and 22 tRNAs (Fig. 1 and Table 1). The length and gene organization of our mitogenome are similar to the first *A. gigas* mitogenome sequenced^11^ with the exception of the NAD5 gene. The NAD5 gene obtained in our mitogenome has 1,728 bp with a complete stop codon (TAA) which is consistent with the length and stop codon (TAA or TAG) of other Strombidae species, while Márquez et al.^11^ obtained a NAD5 length of 1,753 bp with an incomplete stop codon (T–) which is not very common in Hyspogastropoda. Gene order in our mitogenome is similar to other Strombidae^11,14,15,37,38^. This is not surprising considering that gene organization is relatively stable throughout the Gastropoda. When rearrangements do occur in Gastropods, they principally occur in the tRNA^12^. An exception is the Vermetidae which present high levels of gene order rearrangement^39^. The total length of all genes in our mitogenomic sequence represents 97.8% of the length of mitogenome (equivalent to 15,117 bp with: PCGs = 11,259 bp; rRNAs = 2,367 bp; tRNAs = 1,491 bp), and all non-coding regions accounted for 343 bp. Most intergenic regions are very short (< 15 bp), but two larger intergenic regions were identified, one upstream of the COX3 gene (54 bp; between tRNA-Phe and COX3) and one downstream of the COX3 gene (41 bp; between COX3 and tRNA-Lys). Non-coding regions around the COX3 gene have been proposed as candidates for D-loop in other Gastropods^40^ and are characterized by AT-rich content^41^. High AT content (82%) was observed for the non-coding region downstream of the COX3 gene. Three overlaps between adjacent genes were found in our *A. gigas* mitogenome (Table 1) as was identified for *T. dentatus*^15^. Other Strombidae species have eight or six overlaps (*S. pugilis* and *M. variabilis* respectively^15^), four overlaps (*L. lambis* and *H. chiragra*^14^, and *L. canarium*^37^), or only one overlap (*C. luhuanus*^38^). Localization of overlaps into the mitogenome is relatively stable among Strombidae species.

**Table 1:**
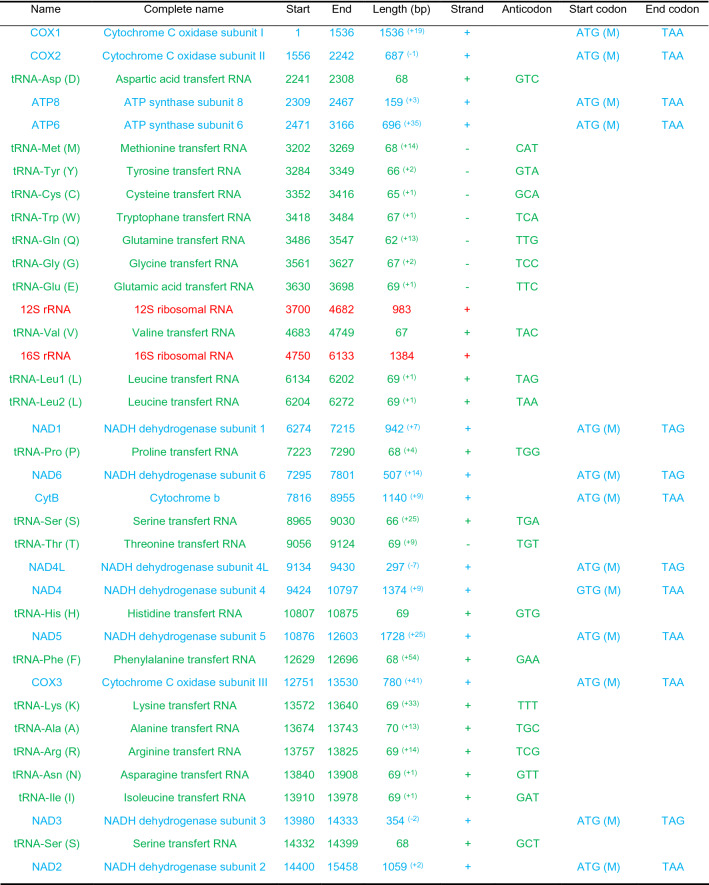
Mitogenome profile of *A. gigas*. The 13 Protein coding genes (PCGs) in blue, the 22 transfer RNA (tRNA) in green, and the two ribosomal RNA (rRNA) in red. All tRNAs have the three and one letter code, numbers in brackets in length column represent the number of nucleotides separating two genes (+) or overlapping two genes (−) downstream from the gene where it is indicated.

Nucleotide composition of our *A. gigas* mitogenome is AT-rich (65.8%) which is consistent with other Strombidae species (Supplementary Table S2), and with the Hypsogastropoda species used in this study (from 60% as for *Dentropoma* sp. to 73% as for *Naticarius hebraeus*). The nine Strombidae mitogenomes analyzed here show an important bias to T over A (AT skew < 0) and a small bias to G over C (GC skew > 0), though this pattern is less pronounced in *C. luhuanus* (note that *Ministrombus variabilis* has an incomplete genome; Supplementary Table S2). Including only PCGs or tRNAs, the nine Strombidae genomes show a similar value of AT content (65–69%), while the two rRNAs have a lightly higher value (67–70%) (Supplementary Table S2). The AT skew is negative and large for the PCGs (from − 0.16 to − 0.20), but positive and less pronounced for rRNAs (< 0.08) and tRNAs (< 0.04). PCGs have little to no GC bias, with very low positive (GC skew < 0.03) or negative (GC skew =  − 0.01) values, while rRNAs and tRNAs show a disequilibrium in the use of G/C in favor of G (GC skew >  > 0) (Supplementary Table S2).

The heavy strand encodes for the 13 PCGs, two rRNAs, and for 14 tRNAs (tRNA-Asp, tRNA-Val, tRNA-Leu1, tRNA-Leu2, tRNA-Pro, tRNA-Ser(TGA), tRNA-His, tRNA-Phe, KARNI complex, and tRNA-Ser(GCT)). The light strand encodes for eight tRNAs, the MYCWQGE complex and tRNA-Thr (which can be located on either strand depending on the species^12^). This genic organization between both strands is constant among Strombidae species. Both strands on our mitogenome are AT-rich (heavy strand with 65.5% and light strand with 67.5%), but the heavy strand shows an important use of T over A (AT skew =  − 0.136) and low use of G over C (GC skew = 0.037), while the light strand shows low use of A over T (AT skew = 0.056) and high use of G over C (GC skew = 0.121). This disequilibrium in the content of A/T and G/C highlights a strand bias potentially demonstrating a difference in mutation rate and/or selective pressure between each strand^42^. The GT-rich composition on the heavy strand is particularly diagnostic for marine bivalves, but the asymmetric mutation pattern between strands could lead to an AC-rich content on the light strand^43^. Our results with *A. gigas*, however, uncover AG-rich content on the light strand exhibiting G-rich content for both strands. G-rich content for the light strand has been identified for the majority of Hypsogastropoda analyzed in this study (though not in *Buccinum pemphigus*, *Nassarius hepaticus*, and *Penion maximus*).

The start and stop codons of the PCGs show variation among Strombidae species (Supplementary Table S3). For our *A. gigas* mitogenome, 12 of the 13 PCGs initiate with ATG (NAD4 starts with GTG) which is the most common start codon in Strombidae, and in gastropods in general^12,14,15,22,38,44^. The PCGs that present the highest start codon variation among Strombidae are NAD4 followed by NAD2, NAD4L, CytB, and ATP6, which have been reported as variable in other species of gastropods (review in^22^). For our *A. gigas* mitogenome, the majority of PCGs end with a TAA stop codon and four ends with TAG (NAD1-NAD6-NAD4L-NAD3). These stop codons are the most common in gastropods^12,14,15,22,38,44^. Considering the Strombidae species analyzed here, nine PCGs show variation among species (Supplementary Table S3).

The use of synonymous codons in the 13 PCGs varies among Strombidae species (Supplementary Fig. S1) and between the two *A. gigas* mitogenomes. Such a pattern could be considered non-random because some codons are used more than others^13^. The five most frequently used codons (with larger RSCU values) for *L. lambis*, *H. chiragra*, *C. luhuanus*, *L. canarium*, *M. variabilis*, and *T. dentatus* are Leu2 (UUA), Ser2 (UCU), Arg (CGA), Ala (GCU), and Pro (CCU) (codon order is species dependent) as reported for other gastropods^13^, while *Strombus pugilis* has Thr (ACU) instead of Arg (CGA). The two mitogenomes of *Aliger gigas*, however, present two different codons in the top 5 frequently used codons: Thr (ACU) and Val (GUU) (order is specimen dependent) instead of Arg and Pro. Leu (UUA), Ser (UCU), and Ala (GCU) are the most frequently used codons reported for gastropods^13,45,46^. The codons least frequently used (lower RSCU values) for both *A. gigas* specimens were Ser2 (UCG) and Thr (ACG), whereas the other Strombidae species presented variation: *L. lambis* [Arg (CGC) and Ser2 (UCG)], *H. chiragra* [Ala (GCG) and Leu1 (CUG)], *C. luhuanus* [Ala (GCG) and Thr (ACG)], *T. dentatus* [Leu1 (CUG) and Pro (CCG)], *S. pugilis* [Ala (GCG) and Arg (CGC)], and *L. canarium* [Ser2 (UCG) and Leu1 (CUG)]. Generally, these codons are reported less frequently in other gastropods as well^13,45,46^ except for Thr (ACG). *Ministrombus variabilis* is unique with Ala (GCG) and stop (UAG) codons as less frequently used. As shown previously in other gastropods^45,46^, the codons rich in A and T are used more frequently in all mitogenomes analyzed here than codons with C or G content, and codons with A or T at the third position are even more utilized (RSCU from 0.89 to 2.62) than those with C or G (RSCU from 0.07 to 0.98).

### Intra- and interspecific variation in Strombidae

The majority (82%; Supplementary Table S4) of SNPs among both *A. gigas* mitogenomes occur in the PCGs, which is similar to the number of SNPs observed at the intraspecific level in other marine species^32,47,48^. A similar pattern emerges at the interspecific level, 80% of SNPs occur in the PCGs among the eight Strombidae species. Few indels were identified for *A. gigas*, a pattern that has been previously demonstrated in other marine species^32,47^. Indels are more numerous at the interspecific level; but, the number of indel events remain very low in the PCGs which is further consistent with a previous study that suggests that indels largely decrease in coding regions^49^. All indels at the interspecific level are registered in the *C. luhuanus* mitogenome and are at the beginning or at the end of genes suggesting a possible bias in its annotation procedures.

At the nucleotide level, both *A. gigas* mitogenomes present high levels of divergence with a nucleotide diversity of 0.0074 considering the whole mitogenome (0.0084 for the PCGs concatenated; Supplementary Table S4). This level of divergence suggests genetic isolation between the Mexican and Colombian lineages of *A. gigas*. The wide geographic distribution of *A. gigas* could permit these levels of genetic divergence through population fragmentation and/or adaptation to local environments as previously suggested in an oyster mitogenome analysis (π = 0.0068)^32^. Also, Galván-Tirado et al.^48^ identified similar genomic divergence between two individuals of sablefish and suggest the presence of two distinct lineages in the northeast Pacific. Our result confirms that *A. gigas*, as a species, does not present a panmictic structure (reviewed in^1^) but rather is comprised of highly structured populations across its geographic range. At the interspecific level, the eight Strombidae species present a higher nucleotide diversity (0.1698 for whole genome and 0.1778 considering the PCGs). These values between species are in the same order of magnitude as for species of Naticidae (Caenogastropoda, Littorinimorpha)^50^. Pairwise species divergence (Table 2) demonstrates a close relationship between *A. gigas* and *Strombus pugilis*, which is consistent with the past generic affiliation of *A. gigas* (previously *Strombus gigas*). Such close affiliation has been identified based on H3 and COI genes^33^ and phylogenomic work^15^. Table 2 further highlights genetic proximity for a clade of three species (*Tridentarius dentatus*, *Ministrombus variabilis*, and *Laevistrombus canarium*).

**Table 2:**
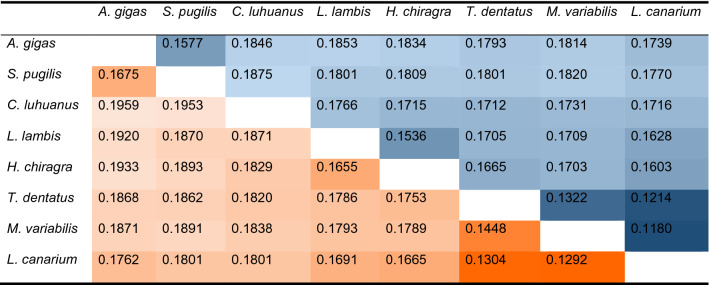
Nucleotide divergence (*k*) for pairs of Strombidae species used in this study using complete mitogenomes (above diagonal, blue color) and the 13 PCGs (below diagonal, orange color). Colors broadly correspond to values of *k,* where darker color represent the lowest values of nucleotide divergence suggesting a closer relationship between species. *Aliger gigas* (*A. gigas*), *Strombus pugilis* (*S. pugilis*), *Conomurex luhuanus* (*C. luhuanus*), *Lambis lambis* (*L. lambis*), *Harpago chiragra* (*H. chiragra*), *Tridentarius dentatus* (*T. dentatus*), *Ministrombus variabilis* (*M. variabilis*), and *Laevistrombus canarium* (*L. canarium*).

**Figure 2:**
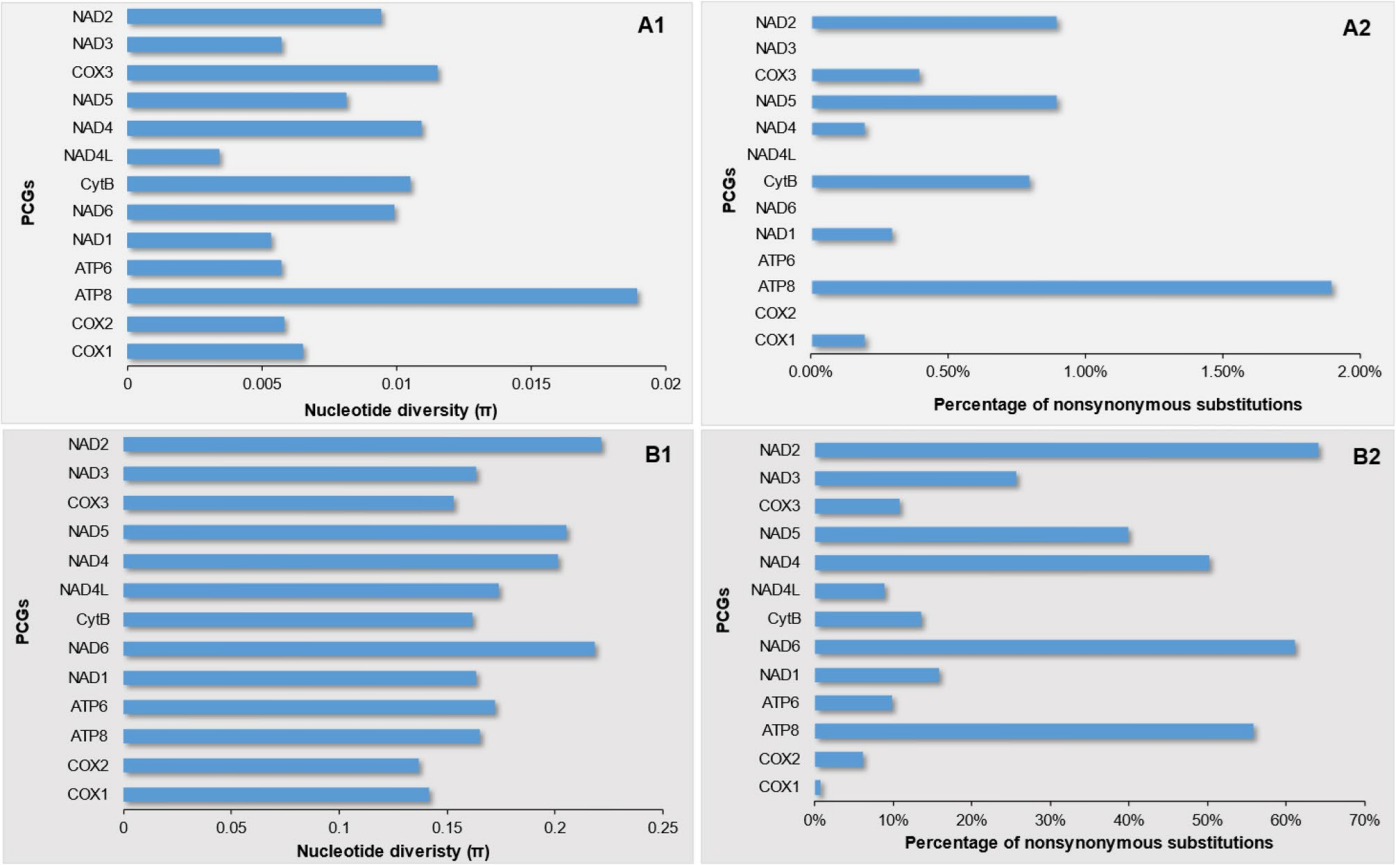
Intra- and interspecific variation in Strombidae. (**A**) intraspecific (*Aliger gigas*), (**B**) interspecific (Strombidae species), (1) nucleotide diversity, (2) proportion in amino acid change per protein coding genes.

The intraspecific diversity at the PCG level (Fig. 2-A1) shows that ATP8 exhibits the highest variation between the two mitogenomes of *A. gigas* followed by COX3, NAD4, CytB, NAD2, and NAD6. NAD4L shows the lowest genetic variation. These results help evaluate which PCGs are best to resolve population genetic issues in the Strombidae. For example, COX1, which present an intermediate value of genetic variation (Fig. 2-A1), has been previously used to resolve population genetic structure in *A. gigas*^34^ but our data suggest that this gene is probably not the best to resolve such population level questions. Furthermore, the proportion of nonsynonymous substitutions (changes in nucleotides that lead to a change in AA) for the PCGs at the intraspecific level ranges from 0% (COX2, APT6, NAD6, NAD3, NAD4L) to almost 2% (ATP8) (Fig. 2-A2). These low values are consistent considering that the rate of amino acid substitution is related with the intensity of selection^13^. Interspecific diversity at the PCG level (Fig. 2-B1) showed very high values for NAD complex genes (NAD1-6 and 4L) and lower values for COX genes (COX1-3) and CytB. Previous work on strombid species relationships used a COX1 fragment^33^, however, our results suggest that the genes belonging to the NAD complex could be more useful in resolving inter-genus phylogenetic relationships^51^. The proportion of nonsynonymous substitutions for the PCGs at the interspecific level are higher (> 50%) for some genes from the NAD complex (NAD2, NAD4-6) and for ATP8 (Fig. 2-B2) and very low for COX1 gene. Such changes to AA in a particular gene can be informative about the impact of purifying or positive selection^32^. As shown for gastropods and other marine species, genes with higher AA substitutions, as seen here in the NAD complex/ATP8, are subjected to stronger positive selection, while highly conserved genes, like the COX complex, are under stronger purifying selection^13,32,47^.

### Phylogeny

**Figure 3:**
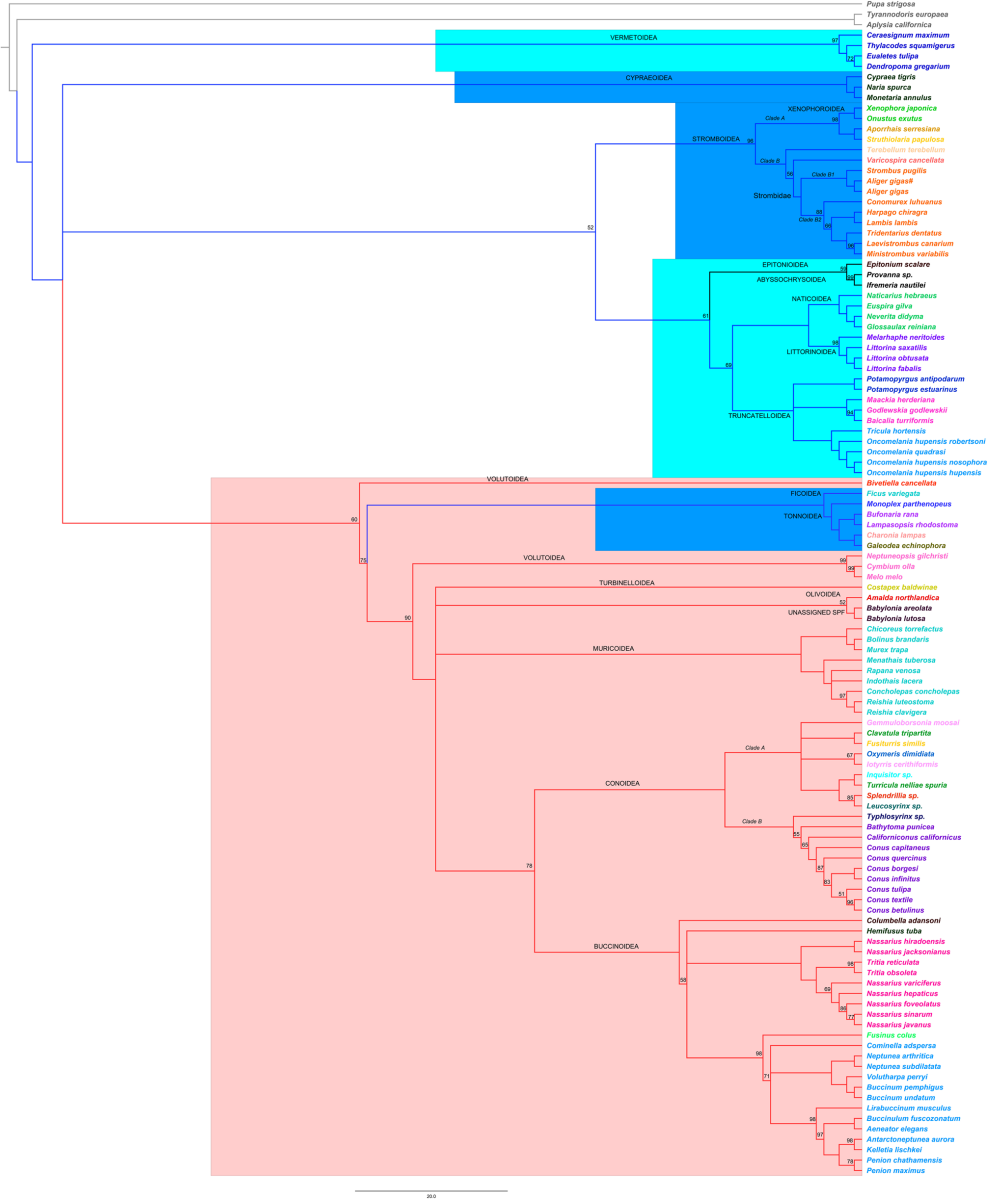
Maximum likelihood phylogenetic tree based on the concatenated nucleotide alignment of the 13 PCGs of 110 Hypsogastropoda mitogenomes. Numbers above branches indicate bootstrap values (branches without a number have a bootstrap of 100). Branch colors follow the classification of Bouchet & Rocroi (2005): Littorinimorpha (blue), Neogastropoda (red), outside both clades (black). Clades proposed by Bouchet et al. (2017) are highlighted: Latrogastropoda (red), Latrogastropoda “uncertain position” (dark blue), Non-Latrogastropoda (light blue). Names of SPFs labeled on branches, and species names are organized and colored by family. The # symbol signals specimen of *A. gigas* sequenced in this study. Three Heterobranchia species were used as outgroups (*Pupa strigosa*, *Aplysia californica*, and *Tyrannodoris europaea*). See Table S1 for sources.

Our genomic study highlights that the large Hypsogastropoda clade is a very complex taxonomic group for which many taxonomic representatives need to be included to elucidate relationships. We present the most complete phylogenomic understanding of the Hypsogastropoda to date including 17 superfamilies, 39 families, 85 genera, 109 species and 110 individuals (Fig. 3). Overall, relationships across the tree are very well supported.

#### SPFs of uncertain position

The increased sampling across the Hypsogastropoda allows us to provide some resolution for various taxonomic uncertainties. The Hypsogastropoda clade was initially divided into two major groups, Littorinimorpha and Neogastropoda^17^. Hypsogastropoda was recently revised by Bouchet et al.^18^ who suggested, instead, that the superorder Latrogastropoda included all Neogastropoda and six SPFs from Littorinimorpha (Calyptraeoidea, Cypraeoidea, Ficoidea, Stromboidea, Tonnoidea, and Xenophoroidea) of “uncertain position”. All other Littorinimorpha SPFs were regrouped as Non-Latrogastropoda^13^. Of the six SPFs that Bouchet et al.^18^ moved from Littorinimorpha to Latrogastropoda but couldn’t otherwise place, five have complete mitogenomes in GenBank (Cypraeoidea, Ficoidea, Stromboidea, Tonnoidea, and Xenophoroidea). The sixth SPF, Calypteraeoidea, has one incomplete mitogenome available (*Calyptraea chinensis* 8,530 pb, EU827193^52^). We decided not to include this mitogenome in our phylogeny as only five of the 13 PCGs were identified. Our results confirm, although with moderate support, that Stromboidea and Xenophoroidea SPFs belong to the Littorinimorpha as proposed originally^17^. Such a relationship has been supported previously by genetic, genomic, and morphological studies^15,16,19,25,26,53,54^. Other investigations have suggested instead that Stromboidea is sister to Cypraeoidea^12,55^ but with low support, or to Tonnoidea^14,23^. Alternative topologies clustering Stromboidea with SPF other than Xenophoroidea are probably due to the absence of representative Xenophoridae, highlighting the importance to include as many SPFs as possible in phylogenetic analyses.

Considering the Stromboidea clade, our results strongly support a monophyletic lineage including the Xenophoridae family (unique living family of Xenophoroidea and represented here by *Xenophora* and *Onustus*) confirming recent genomic work^15^ and, previous behavioral and morphological studies (reviewed in^15^). The well-supported Stromboidea clade is divided into two clades. Clade A suggests that members of family Xenophoridae are sister to representatives of Aporrhaidae + Struthiolarridae. Although this topology is not known to be supported by morphology^53^ such a topology has been similarly recovered^15^. Clade B is comprised of three groups, with one representative of Seraphsidae (*Terebellum*) resolved as sister to members of Rostellariidae (*Varicospira*) + Strombidae. Recent morphological work^56^ supports these major clade assignments (Aporrhaidae + Struthiolarridae separate from the Seraphsidae + Rostellariidae + Strombidae). Within Strombidae (in orange in Fig. 3), we obtained a topology identical to Irwin et al.^15^, though we further clarify the placement of one additional genus (*Tridentarius*). We identify two principal clades: Clade B1 strongly confirms the sister relationship between *Strombus* and *Aliger* (*Aligerina* and *Strombina* clades in Fig. 3;^57^), and clade B2 is comprised of six genera. *Conomurex* is the earliest diverging lineage of this clade and sister to two clades (*Harpago* + *Lambis*) and (*Tridentarius* + (*Laevistrombus* + *Ministrombus*)). The clade formed by *L. lambis* and *H. chiragra* (syn. *L. chiragra*^33^) determined by mitogenomes is largely accepted^14,15,33,58^. Clade B1 and B2 represent biogeographically structured clades as previously noted^33^: An Eastern Pacific/Atlantic clade to which *Aliger* (syn. *Strombus*) *gigas* and *Strombus puglis* belong (clade B1), and an Indo-West Pacific clade with *Lambis*, *Harpago*, *Conomurex, Tridentarius*, *Laevistrombus*, and *Ministrombus* (clade B2).

Though we clarify some relationships in Strombidae, as outlined above, and as confirmed by our measures of interspecific variation (Table 2), phylogenetic relationships between strombid genera remain controversial. For example, morphological and genetic studies in the two most species-rich genera, *Lambis* and *Strombus,* suggest different patterns. *Lambis* has been proposed as monophyletic^33^ nested within a paraphyletic *Strombus*^33,54^, while an older morphological study^58^ proposed *Lambis* as paraphyletic and *Strombus* as polyphyletic. The topology inside Strombidae obtained using mitogenomes (this study and^14,15^) has not been supported previously by morphology^53^ or genetic studies^33^. Future work should aim to use mitogenomes with increased species sampling and including nuclear genes to explore relationships among these species rich and difficult to resolve genera.

The phylogenetic affinity of SPF Cypraeoidea remains unresolved in our study with respect to its membership in Littorinimorpha or Neogastropoda. Thus, we offer no resolution to its historically uncertain phylogenetic position in the Hypsogastropoda^12,15,16,54,55,59^. Our genomic study resolved the phylogenetic position of Tonnoidea and Ficoidea SPFs. The placement of Tonnoidea as an early branching lineage within the Neogastropoda clade is largely confirmed^12,13,15,16,55,59^. The inclusion of the mitogenome of Ficoidea SPF (*Ficus variegate*;^60^) confirms that Tonnoidae is sister to Ficoidea as recently suggested^26^. Though some studies have considered Tonnoidae as the sister clade to the Cancellariidae (represented by *Bivetiella cancellata*;^13,59^), our results do not confirm this relationship (see discussion below). Within Tonnoidae, the inclusion of a new mitogenome (*Charonia lampas*) highlights that Ranellidae (now Charoniidae (*Charonia* sp.)) + Cymatiidae (*Monoplex* sp.) is paraphyletic and supports previous studies^54,59^. Our work confirms the ultimate Tonnoidea relationships based on genetic data^26^ whereby Charoniidae (here represented by *Charonia lampas*) are sister to Cassidae (here represented by *Galeodea echinophora*), which in turn are sister to Bursidae (here represented by *Bufonaria rana* and *Lampasopsis rhodostoma*). This clade is in turn sister to Cymatiidae (here represented by *Monoplex parthenopeus*).

#### Non-Latrogastropoda

Within the Non-Latrogastropoda, the addition of the *Epitonium scalare* mitogenome suggests SPF Epitonioidea as sister to Abyssochrysoidea with high support. The clade (Epitonioidea + Abyssochrysoidea) is further resolved as sister to three Littorinimorpha SPFs (Littorinoidea, Naticoidea, Truncatelloidea). SPF Abyssochrysoidea has been previously reported as sister to the majority of Littorinimorpha^12,13^ or to Vermetoidea^15^ with low support, these proposed positions might be due to the absence of mitogenomes from the SPF Epitonioidea. Morphological work^61^ that suggested that Epitonioidea is sister to Neogastropoda is refuted in our genomic reconstruction. The phylogenomic relationships among the remaining Littorinimorpha in this clade show a well-supported SPF Truncatelloidea (named Rissoiform clade; Supplementary Table S1), as previously proposed^18^, that is sister to a well-supported Littorinoidea + Naticoidea clade which has also been recovered in previous genomic studies^14,22^. Our genomic study did not support Truncatelloidea and Littorinoidea + Naticoidea as early branching lineages of Hypsogastropoda^15,59^.

The SPF Vermetoidea, placed as sister clade to the rest of Caenogastropoda, is well-supported in our tree and has been largely demonstrated elsewhere e.g.,^12,13,55^. However, other phylogenomic work has suggested that Vermetoidea should be sister clade to a clade formed by the subclasses Caenogastropoda + Neritimorpha + Vetigastropoda^62^, or that Vermetoidea is sister clade to Abyssochrysoidea^15^. Mitochondrial gene rearrangement could explain the controversial phylogenetic position of Vermetoidea^39^. Gene rearrangement has been associated with higher rates of nucleotide substitution and is observed as long-branches in phylogenetic trees^12,13^. Fourdrilis et al.^13^ suggested that the relationship between the gene order rearrangement rate and the nucleotide substitution rate might not apply for all Caenogastropoda. Mitochondrial gene order rearrangement is very common in many taxonomic groups^63–65^ but very few studies go on to explain the possible biological reasons for extensive rearrangement in mitochondrial genes. Lockridge and Boore^65^ suggested that selection at the organismal level might select for mitochondrial gene rearrangement. Vermetoidea present a unique lifestyle when compared to other Caenogastropoda considering that they are one of only two lineages that cement their shell directly to hard substrates and live a sessile life, the other group is the freshwater *Helicostoa*^25,39^. We hypothesize that adaptive selection on the Vermetoidea lifestyle acted at both the organismal and cellular levels. More mitogenomic data as well as structural and functional genomic studies related to nuclear DNA will be necessary to understand the biological implications of mitochondrial gene rearrangement and to further clarify the phylogenetic position of the Vermetoidea.

#### Latrogastropoda

The backbone of the Latrogastropoda clade (highlighted in red on Fig. 3) places one species of Volutoidea, *Bivetiella cancellate*, as sister to the remainder of the group and subsequently identifies Volutoidea as not monophyletic. Furthermore, our genomic study confirms the inclusion of Ficoidea and Tonnoidea SPFs in Latrogastropoda as suggested previously^18^. *Bivetiella cancellata* (Cancellariidae, SPF Volutoidea) as sister to the rest of the clade has been proposed before^12,62^ though sometimes in association with other SPFs, for example with Calyptraeoidea^15^ or Tonnoidea^22,59^. Previously classified in Cancellarioidea SPF, the Cancellariidae was recently incorporated into Volutoidea SPF based on a recent phylogenetic analysis^66^ and used in the classification of Bouchet et al.^18^. Phylogenomic analyses^12,13,15,22,55,59^, however, demonstrate the separation of *B. cancellata* from the rest of Volutidae, suggesting that Cancellarioidea and Volutoidea must be considered as separate SPF as also supported by our study. Turbinelloidea is unresolved in our phylogenomic tree though it was considered as sister to a clade formed by Olivoidea + Muricoidea + Babyloniidae (unassigned SPF) + Buccionoidea + Conoidea^67^. Babyloniidae, an unassigned SPF, is sister to SPF Olivoidea with moderate support as previously proposed^13^ though its relationship to other SPFs is uncertain on our tree. Yang et al.^45^ proposed Babyloniidae as sister to the Buccinoidea but didn’t include Olivoidea in their study. We increased the number of species of Buccinoidea (n = 16) included in a phylogenomic framework and we included two additional families (Melongenidae and Fasciolariidae) in our analysis. Buccinoidea families with more than one representative are monophyletic in our reconstruction, though phylogenetic relationships between several families is uncertain. Family Columbellidae (represented by *Columbella adansoni*) is supported as the earliest diverging lineage in the SPF as previously suggested^13^, Melongenidae (*Hemifusus* sp.) presents an unresolved situation in the clade, and Fasciolariidae (*Fusinus* sp.) is confirmed as sister clade to Buccinidae^68^. Sister to the Buccinoidea is SPF Conoidea. Our tree considers a high number of specimens (n = 11; eight species and four families) representing SPF Conoidea. Our reconstruction highlights a complex situation for this SPF as recently suggested^69^. Two distinct clades are observed and correspond to those previously identified^69^: clade A includes three paraphyletic families (Turridae, Clavatulidae, and Pseudomelomitidae) as well as Drilliidae and Terebridae, and clade B includes all Conidae species that are sister to Raphitomidae (represented here by *Typhlosyrinx* sp.). These results emphasize and support the need to sequence more mitochondrial genomes to improve resolution within SPF Conoidea^69^.

### Divergence times

**Figure 4:**
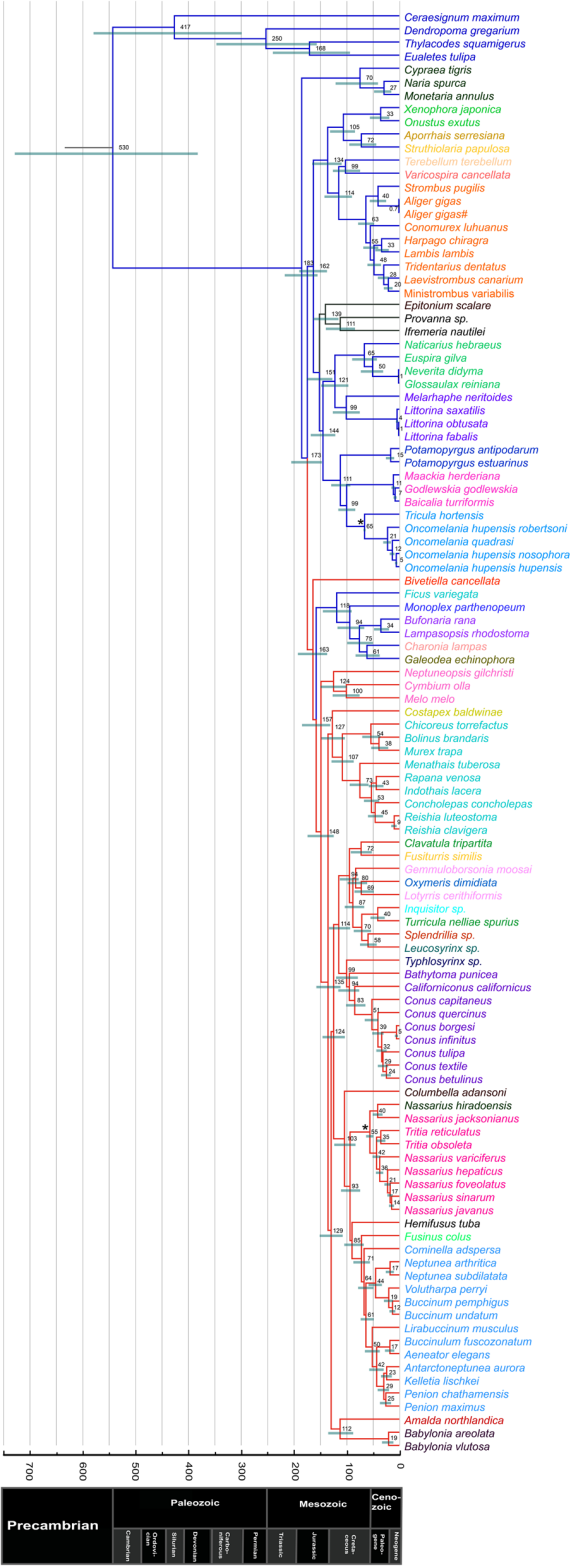
Estimates of divergence time inferred from Bayesian analysis of the 13 PCGs of 110 Hypsogastropoda mitogenomes. Branch colors represent classification following Bouchet & Rocroi (2005): Littorinimorpha (blue), Neogastropoda (red), outside both clades (black). Branch lengths are proportional to time (in Mya). Node values represent posterior mean ages and green bars indicate the 95% HPD. Black asterisks indicate calibrated nodes (see methods). Outgroups have been trimmed. See Table S1 for sources.

The reconstruction of a divergence time tree dates the Strombidae (in orange on Fig. 4) diversification to the Paleocene (63 Mya; 95% HPD: 47.6–78.0). Previous work^27^ similarly suggested that Strombidae originated at the end of the Cretaceous with a rapid diversification at early Cenozoic. In their genomic study, Jiang et al.^14^ identified the origin of Strombidae at late lower Cretaceous (112 Mya; interval: 44–206 Mya), however, Bandel^24^ suggested a much more recent origin of the Strombidae (Oligocene: 33.9–23.0 Mya). Within the Strombidae, our divergence time estimates suggest that *Lambis* and *Harpago* diverged around 33 Mya which is close to a previous estimate (~ 23 Mya)^27^. Jiang et al.^14^, however, estimated the divergence between these genera to the Eocene. The earliest branching lineage of clade B2 (Fig. 3), *C. luhuanus*, is estimated to have diverged from the other species 55 Mya (late Eocene). Divergence between *Strombus* and *Aliger* (clade B1 in Fig. 3) was estimated at 40 Mya (Eocene) on our chronogram which coincides with the proposed pre-Miocene common ancestry between *Aligerina* and *Strombina*^57^.

Our analyses suggest recent lineage divergence within *Aliger gigas* during the Pleistocene (0.7 Mya). Generally, few data have been available for lineage divergence; nevertheless, our Pleistocene estimate could be considered as very recent when compared with a genetic study^26^ that showed divergence between relatively geographically close specimens within species estimates of Tonnoidea to be much older (from 1.52 Mya to 6.24 Mya).

We estimate that the two principal clades of Stromboidea (clade A: Xenophoridae + Aporrhaidae + Struthiolarridae and clade B: Seraphsidae + Rostellariidae + Strombidae; Fig. 3) diverged around 134 Mya (early Cretaceous) which is similar to the other estimates (153 Mya)^26^; though another study suggested a much older origin (Triassic) for Stromboidea^24^. Our analyses suggested divergence between Xenophoridae and clade Aporrhaidae + Struthiolarridae at 105 Mya (Mid-Cretaceous) which is consistent with the proposed emergence of Aporrhaidae during the Mid-Cretaceous^24^ while Roy^27^ estimated that Aporrhaidae evolved during the latest Triassic (~ 200 Mya). Our tree estimated diversification of the Struthiolariidae at 72 Mya while Bandel^24^ indicated that this family evolved in the early Tertiary (~ 66 Mya). The divergence times between Rostellaridae and Seraphisidae is estimated at 99 Mya, which is consistent with previous Rostellaridae estimates^24^.

Our analyses estimated the divergence time of the Vermetoidea from the rest of Hypsogastropoda during the early Paleozoic (530 Mya) which is relatively close to other estimates (423 Mya)^14^. Yang et al.^45^, however, estimated the divergence of these groups much more recently (137 Mya). We estimated the diversification of Hypsogastropoda (excluding Vermetoidea) at 183 Mya (mid-Jurrasic) which is consistent with the literature^16^ that mentions some Hypsogastropoda families in the mid-Jurrasic and a few representatives in the Paleozoic. We estimate the divergence of the SPF Tonnoidea at 118 Mya which is more recent than another study [186 Mya; 26]. Our genomic dating evaluation of divergence time among Tonnoidae families is generally earlier than those proposed by Strong et al.^26^, but more in accordance with other work^70^.

## Methods

### Specimen collection, DNA extraction, and sequencing

The individual of *Aliger gigas* used for this study comes from the Cozumel Island Protected Area of Fauna and Flora (Mexico) and was received in 2013 from the relevant authority of the park following a seizure of illegal catch. Sample tissue was preserved in 96% ethanol and maintained at 4 °C until extraction. Total genomic DNA was extracted using the EZNA DNA purification kit (Omega Bio-Tek, Norcross, GA). DNA libraries were constructed by shearing the DNA on a Bioruptor Illumina TruSeq (Illumina, San Diego, CA) with compatible adapters and custom indices using Kapa BioSciences library preparation kits (KapaBiosystems, Woburn, MA). Library quality was checked, normalized, pooled, and run on an Illumina MiSeq (paired-end 250 reads, Illumina, San Diego, CA) at the Georgia Genomics Facility (University of Georgia).

### Genome assembly and annotation

The quality of sequence reads was evaluated using FastQC (http://www.bioinformatics.babraham.ac.uk/projects/fastqc/). Adapters and low-quality read ends (Phred score < 20) were removed manually in Geneious 11.1.3 (http://www.geneious.com/). The *A. gigas* mitogenome was reconstructed by mapping reads to the *Aliger gigas* reference genome (NC024932)^11^ in Geneious 11.1.3. The 13 PCGs were identified and annotated using MITOS^71^ and DOGMA^72^, while the tRNA genes were identified using tRNAscan-SE 2.0^73^ and ARWEN 1.2^74^. The rRNA genes were identified and annotated by comparing the MITOS results, Blastx information, and the reference mitogenome. Finally, our *A. gigas* mitogenome map was visualized using GenomeVx^75^.

### Sequence analysis and genomic diversity

Analysis of nucleotide composition, including AT content, was assessed using Geneious Prime 2019.0.4; nucleotide skew (nucleotide bias) statistics were determined by AT skew (AT skew > 0 means A-rich and AT skew < 0 means T-rich) and GC skew (GC skew > 0 means G-rich and GC skew < 0 means C-rich)^76^. Nucleotide skew analyses were conducted for the nine Strombidae mitogenomes (Supplementary Table S1) considering the whole mitogenome, the 13 concatenated PCGs, the 22 concatenated tRNA, the two concatenated rRNA, and the heavy and light strands. The RSCU was determined using Mega X^77^ for each of the 13 PCGs from the nine Strombidae mitogenomes.

Parameters of intra- and interspecific variation within Strombidae were assessed at three levels: whole mitogenomes, the 13 concatenated PCGs, and for each of the 13 PCGs. At each level, target sequences from each species were aligned using MAFFT 7.450^78^ and the sequences from our *A. gigas* mitogenome was used as the reference. The total number of SNPs, indel sites, and indel events were determined using DnaSP 6.10.03^79^ and manually checked in Geneious Prime. The number of synonymous and nonsynonymous substitutions were identified in Geneious Prime. Finally, nucleotide diversity (*π*) for each species pair, and nucleotide divergence (*k*), were determined using DnaSP 6.10.03 considering the whole mitogenome and the 13 concatenated PCGs.

### Phylogenetic inference and divergence time analyses

Phylogenetic analyses were performed with a total of 110 complete or partial mitogenomes in clade Hypsogastropoda downloaded from GenBank (up to January 2021) including our *A. gigas* mitogenome, representing 109 species, 85 genera, 39 families, and 17 superfamilies (Supplementary Table S1). Three species belonging to the Heterobranchia clade were used as outgroups: *Pupa strigosa* Gould, 1859 (Acteonidae), *Aplysia californica* J.G. Cooper, 1863 (Aplysiidae), and *Tyrannodoris europaea* García-Gómez, 1985 (Polyceridae). Phylogenetic analyses were performed with nucleotide sequences using the 13 concatenated PCGs. A saturation analysis was performed in Dambe 7.2.43^80^ and no saturation was observed at the node including all Hypsogastropoda (except SPF Vermetoidae). The third codon position was retained in our dataset^81,82^. Nucleotide sequences were aligned in MAFFT 7.450, and ambiguously aligned positions were removed with GBlocks 0.91b^83^. The best-fit models of nucleotide substitution were evaluated using jModelTest 2.1^84,85^ considering the Bayesian information criteria^86^. Two models were tested (GTR + I + G and GTR + G) and the best fit model was selected for final analyses.

Phylogenetic relationships were inferred using the ML method^87^ and conducted with RAxML 8.2.11^88^ implemented in Geneious Prime using the GTR + G nucleotide substitution model, and rapid bootstrapping using a rapid hill-climbing algorithm. Branch support was evaluated with 1000 bootstrap replicates. The majority consensus tree was constructed considering a 25% of burn-in. Range of branch support were defined for ML tree as follows: maximal for 100%, high for ≥ 70%, moderate for 50–69%, and poor for < 50%^12^.

Inference of divergence times using the 113 aligned mitogenomes (including outgroup) was performed in Beast 2.6.3 on the public web server CIPRES Science Gateway v3.3 (http://www.phylo.org/index.php/) with the input file created in Beauti 2.6.3^89^. The best-fit model previously determined (GTR + G) was used under the uncorrelated lognormal relaxed clock and the Yule speciation evolutionary model. The ML phylogenetic reconstruction was used as a starting tree. Three independent runs were processed with a MCMC of 20 million generations sampling every 1,000 generations with 2 million pre-burn-in. LogCombiner 2.5.2 was used to combine the log and tree files from the three independent runs generated in Beast. Tracer 1.5^90^ was used to evaluate the convergence of chains and confirm that the values of effective sample size (ESS) were above 200 for posterior and likelihood parameters as is recommended; finally, the first 15% of trees were discarded from the combined tree file and a maximum clade credibility tree with a posterior probability limit of 0.5 was obtained using TreeAnnotator 2.5.2. The posterior distribution of estimated divergence times was calculated using two calibration points based on fossils. The first calibration point was under a normal prior and set for the divergence of Nassariidae based on the oldest records of *Buccitriton* 51.9 + / − 4.1 Mya^91^. The second calibration point was the oldest fossil record for the Truncatellidae clade at 66.04 Mya^14^,www.fossilworks.org. Finally, visualization of the ML and divergence time trees was performed using FigTree 1.4.4^92^.

## Supplementary Information

Supplementary Information 1.

Supplementary Information 2.

## Data Availability

The new mitochondrial genome of *Aliger gigas* is available at GenBank under the accession number MZ157283.

## Abbreviations

*AA*: Amino acid
*ATP genes*: Genes encoding ATP synthase subunits 6 and 8
*Bp*: Base pairs
*COX genes*: Genes encoding cytochrome C oxidase subunits I-II-III
*CytB*: Cytochrome B
*D-loop*: Control region
HPD: Highest posterior density
*KARNI*: Complex of consecutive tRNA including Lysine-Alanine-Arginine-Asparagine-Isoleucine
*ML*: Maximum likelihood
*Mya*: Millions years ago
*MYCWQGE*: Complex of consecutive tRNA including Methionine-Tyrosine-Cysteine-Tryptophane-Glutamine-Glycine-Glutamic acid
*NAD genes*: Genes encoding NADH dehydrogenase subunits 1 to 6 and 4L
*PCG*: Protein-coding gene
r*RNA*: Ribosomal RNA
*RSCU*: Relative synonymous codon usage
*SNP*: Single-nucleotide polymorphism
*SPF*: Superfamily
t*RNA*: Transfer RNA
