# Supplementary material for: The queen conch mitogenome: intra- and interspecific mitogenomic variability in Strombidae and phylogenetic considerations within the Hypsogastropoda
Source: Sci Rep. 2021 Jun 7;11:11972. doi: 10.1038/s41598-021-91224-0 (PMC8184947; doi:10.1038/s41598-021-91224-0)
Supplement: Supplementary file 1 — Supplementary Information 1. [file 41598_2021_91224_MOESM1_ESM.pdf]

The queen conch mitogenome: intra- and interspecific mitogenomic variability in Strombidae and phylogenetic considerations within the Hypsogastropoda

Salima Machkour-M’Rabet, Margaret M. Hanes, Josué Jacob Martínez-Noguez, Jorge Cruz-Medina, Francisco J. García-De León

Supplementary Table S1. Summary of sample information for Hypsogastropoda used in this study. Higher classification levels (Superfamily and Clade) are presented considering two classifications: Bouchet & Rocroi (2005) (left) and Bouchet *et al.* (2017) (right). Nomenclature of species and families have been updated following MolluscaBase (https://www.molluscabase.org/). To facilitate comparison with previous studies, previous names are provided below table (*[3]). GenBank acc. nb. (accession number in GenBank database), Ref (reference for the genome publication; Unpub means no publication is related with this genome), Size (total length of the mitogenome).

Follow Bouchet & Rocroi 2005		Family	Species	GenBank acc. nb.	Ref	Size (bp)	Follow Bouchet *et al*. 2017	
Clade	Superfamily						Superfamily	Clade
Zygopleuroid Group		Provannidae	*Ifremeria nautilei* ●■¶	NC024642	a	15,664	Abyssochrysoidea	Rissoiform Clade; Non-Latrogastropoda
			Provanna sp. ¶	KM675481	Unpub	16,183		
Ptenoglossa Group		Epitoniidae	*Epitonium scalare* *[2] §	MK251987	b	15,140	Epitonioidea	
Littorinimorpha	Littorinoidea	Littorinidae	*Littorina fabalis* *[1] ■	KU952092	c	16,418	Littorinoidea	
			Littorina obtusata *[1] ■	KU952093	c	16,400		
			Littorina saxatilis ■¶	NC030595	c	16,887		
			Melarhaphe neritoides *[1] §	MH119311	d	15,676		
	Naticoidea	Naticidae	*Naticarius hebraeus* ●■¶	NC028002	e	15,384	Naticoidea	
			Glossaulax reiniana §	NC041162	f	15,254		
			Euspira gilva *[3] §	MK395168	Unpub	16,139		
			Neverita didyma §	MK548644	g	15,252		
	Vermetoidea	Vermetidae	*Ceraesignum maximum* *[3] ●■¶	HM174253	h	15,578	Vermetoidea	
			Dendropoma gregarium ■	HM174252	h	15,641		
			Eualetes tulipa ■¶	HM174254	h	15,078		
			Thylacodes squamigerus ●■	NC014588	h	15,544		
	Rissooidea	Amnicolidae *[4]	*Baicalia turriformis* ■	KY697386	i	15,127	Truncatelloidea *[4]	
			Godlewskia godlewskii ■	KY697387	i	15,224		
			Maackia herderiana ■	KY697388	i	15,154		
		Pomatiopsidae	*Oncomelania hupensis robertsoni* ■	LC276228	Unpub	15,188		
			Oncomelania hupensis hupensis ●■	LC276224	Unpub	15,184		
			Oncomelania hupensis nosophora §	LC276226	Unpub	15,182		
			Oncomelania quadrasi §	LC276227	Unpub	15,184		
			Tricula hortensis ●■	EU440735	j	15,179		
		Tateidae *[4]	*Potamopyrgus antipodarum* ●■	GQ996430	k	15,110		
			Potamopyrgus estuarinus ●■	GQ996415	k	15,120		
	Cypraeoidea	Cypraeidae	*Naria spurca* *[1,3] ●	KP716636	e	11,107	Cypraeoidea	Uncertain position; Latrogastropoda
			Cypraea tigris ¶	MK783263	l	16,177		
			Monetaria annulus ¶	LC469295	fff	16,087		
	Stromboidea	Strombidae	*Conomurex luhuanus* *[3] ■¶	KY853669	m	15,799	Stromboidea	
			Aliger gigas *[3] ●■¶	NC024932	n	15,461		
			Aliger gigas# *[3] §	MZ157283	This study	15,460		
			Lambis lambis ¶	MH115428	o	15,481		
			Harpago chiragra *[3] ¶	MH122656	o	15,460		
			Ministrombus variabilis *[1] ¶	MW244824	aaa	15,292		
			Strombus pugilis ¶	MW244819	aaa	15,809		
			Tridentarius dentatus ¶	MW244820	aaa	15,500		
			Laevistrombus canarium §	MT937083	bbb	15,626		
		Aporrhaidae	*Aporrhais serresiana* ¶	MW244817	aaa	15,455		
		Rostellariidae	*Varicospira cancellata* ¶	MW244822	aaa	15,864		
		Seraphsidae	*Terebellum terebellum* ¶	MW244821	aaa	15,478		
		Struthiolariidae	*Struthiolaria papulosa* ¶	MW244818	aaa	15,475		
	Tonnoidea	Cymatiidae *[4]	*Monoplex parthenopeus* *[3] ●■¶	EU827200	p	15,270	Tonnoidea	
		Charoniidae *[4]	*Charonia lampas* ¶	NC037188	q	15,405		
		Cassidae *[4]	*Galeodea echinophora* ●■¶	NC028003	e	15,388		
		Bursidae	*Bufonaria rana* §	MT408027	ccc	15,510		
			Lampasopsis rhodostoma *[3] §	MW316791	ddd	15,392		
	Ficoidea	Ficidae *[4]	*Ficus variegata* §	MW376482	eee	15,736	Ficoidea	
	Xenophoroidea	Xenophoridae	*Onustus exutus* *[1,2] ¶	MK327366	r	16,043	Xenophoroidea	
			Xenophora japonica ¶	MW244823	aaa	15,684		
Neogastropoda	Muricoidea	Muricidae	*Bolinus brandaris* ●■	EU827194	p	15,380	Muricoidea	
			Concholepas concholepas ●■	NC017886	s	15,495		
			Menathais tuberosa ■	NC031405	t	15,294		
			Rapana venosa ●■	KM213962	u	15,271		
			Reishia clavigera *[3] ●■	NC010090	v	15,285		
			Chicoreus torrefactus §	NC039164	Unpub	15,359		
			Indothais lacera §	NC037221	w	15,272		
			Murex trapa ¶	MN462589	x	15,408		
			Reishia luteostoma *[3] §	NC039165	Unpub	15,301		
		Babyloniidae *[4]	*Babylonia areolata* ●■	HQ416443	Unpub	15,445	Unassignated SP	
			Babylonia lutosa ■¶	NC028628	y	15,346		
		Volutidae	*Cymbium olla* ●■¶	EU827199	p	15,375	Volutoidea	
			Melo melo ¶	MN462590	z	15,721		
			Neptuneopsis gilchristi ¶	MN125492	aa	15,312		
		Costellariidae	*Costapex baldwinae* §	MW044625	ggg	15,321	Turbinelloidea	
	Cancellarioidea	Cancellariidae	*Bivetiella cancellata* *[3] ●■¶	EU827195	p	16,648	Volutoidea	
	Buccinoidea	Buccinidae	*Buccinum pemphigus* ■	NC029373	bb	15,265	Buccinoidea	
			Neptunea arthritica ■	KU246047	cc	15,256		
			Volutharpa perryi ■	NC028183	dd	15,255		
			Aeneator elegans §	NC039120	ee	15,254		
			Antarctoneptunea aurora §	NC039117	ee	15,227		
			Buccinulum fuscozonatum §	NC039121	ee	15,246		
			Buccinum undatum ¶	NC040940	ff	15,265		
			Cominella adspersa §	NC039125	ee	15,251		
			Kelletia lischkei §	NC039123	ee	15,225		
			Lirabuccinum musculus *[1,2] §	MH931230	gg	15,292		
			Penion chathamensis §	NC039116	ee	15,227		
			Penion maximus §	NC037237	Unpub	15,249		
			Neptunea subdilatata *[3] §	MG827217	Unpub	15,393		
		Columbellidae	*Columbella adansoni* ●■	KP716637	e	16,272		
		Nassariidae	*Tritia obsoleta* *[3] ●■	DQ238598	hh	15,263		
			Tritia reticulata *[3] ●■	EU827201	p	15,271		
			Nassarius variciferus *[3] ■	NC029173	Unpub	15,269		
			Nassarius hepaticus §	MH885313	ii	15,732		
			Nassarius foveolatus §	NC041546	jj	15,343		
			Nassarius jacksonianus §	NC041548	jj	15,234		
			Nassarius javanus §	MH346210	jj	15,325		
			Nassarius sinarum *[3] §	NC041545	jj	15,325		
			Nassarius hiradoensis *[3] §	NC037887	kk	15,194		
		Melongenidae	*Hemifusus tuba* §	MN462591	ll	15,483		
		Fasciolariidae	*Fusinus colus* *[3] §	NC045906	hhh	16,319		
	Conoidea	Conidae	*Conus borgesi* *[3] ●	EU827198	p	15,536	Conoidea	
			Californiconus californicus ■	NC032377	mm	15,444		
			Conus capitaneus ■	NC030354	nn	15,829		
			Conus textile *[3] ●■	KX155574	Unpub	15,765		
			Conus tulipa ■	NC027518	oo	15,756		
			Conus betulinus §	NC039922	Unpub	16,240		
			Conus infinitus *[3] §	KY864967	pp	15,522		
			Conus quercinus §	MH400188	qq	16,439		
			Bathytoma punicea §	MH908389	rr	16,037		
		Clavatulidae	*Turricula nelliae spuria* §	MK251986	ss	16,453		
			Clavatula tripartita *[1] §	MH308391	rr	15,743		
		Drilliidae	*Splendrillia* sp. §	NC038184	rr	15,358		
		Terebridae	*Oxymeris dimidiata* *[3] ●■	EU827196	p	16,513		
		Fusiturridae *[4]	*Fusiturris similis* ●■	EU827197	p	15,595		
		Turridae	*Iotyrris cerithiformis* *[3] ●■	DQ284754	tt	15,380		
			Gemmuloborsonia moosai §	NC038183	rr	15,541		
		Pseudomelatomidae *[4]	*Leucosyrinx* sp. §	NC038185	rr	15,358		
		Raphitomidae	*Typhlosyrinx* sp. §	NC038186	rr	15,804		
		Pseudomelatomidae	*Inquisitor* sp. *[1] §	MH308403	rr	15,248		
	Olivoidea	Ancillariidae *[4]	*Amalda northlandica* ●■¶	GU196685	uu	15,354	Olivoidea	
**Outgroup**								
	Acteonoidea	Acteonidae	*Pupa strigosa*	NC002176	xx	14,189		
	Aplysioidea	Aplysiidae	*Aplysia californica*	AY569552	yy	14,117		
	Polyceroidea*[4]	Polyceridae	*Tyrannodoris europaea* *[3]	AY083457	zz	14,472		

● genomes used in the phylogenomic analysis in Osca *et al.* 2015; ■ genomes used in the phylogenomic analysis in Fourdrilis *et al.* 2018; ¶ genomes used in the phylogenomic analysis in Irwin *et al.* 2021; § additional genomes used in this study; #: *A. gigas* determined in this study; ***[1]**: partial genome; ***[2]**: unverified; ***[3]**: correspondence of species names between publication, GenBank register, and/or MolluscaBase (in alphabetic order): *Africonus borgesi* = *Conus borgesi*, *Aliger gigas* = *Lobatus gigas* = *Strombus gigas*, *Cancellaria cancellata* = *Bivetiella cancellata*, *Ceraesignum máximum* = *Dendropoma maximum*, *Conomurex luhuanus* = *Strombus luhuanus*, *Conus infinitum* = *Africonus infinitum*, *Conus textile* = *Cylinder textile*, *Fusinus colus* = *Fusinus longicauda* (*longicaudus*), *Harpago chiragra* = *Lambis chiragra*, *Lotyrris cerithiformis* = *Lophiotoma cerithiformis*, *Lampasopsis rhodostoma* = *Bursa rhodostoma*, *Lunatia gilva* = *Euspira gilva*, *Monoplex parthenopeus* = *Cymatium parthenopeum*, *Naria spurca* = *Erosaria spurca*, *Nassarius hiradoensis* = *Reticunassa hiradoensis*, *Nassarius sinarum* = *Nassarius sinarus*, *Nassarius variciferus* = *Varicinassa varicifera*, *Neptunea subdilatata* = *Siphonalia subdilatata*, *Oxymeris dimidiata* = *Terebra dimidiate*, *Reishia clavigera* = *Thais clavigera*, *Reishia luteostoma* = *Thais luteostoma*, *Tritia obsoleta* = *Ilyanassa obsolete*, *Tritia reticulata* = *Nassarius reticulatus*, *Tyrannodoris europaea* = *Robastra europeae*; ***[4]** Changes in SPF and family names (in alphabetic order): Amnicolidae previously classified in Baicaliidae, Ancillaridae previously classified in Olividae, Babyloniidae previously classified in Buccinidae, Cassidae previously classified in Tonnidae, Cymatiidae and Charoniidae previously classified as Ranellidae, Ficidae previously classified in Tonnoidea, Fusiturridae previously classified in Turridae, Polyceroidea previously classified in Anadoridoidea (SPF), Pseudomelatomidae previously classified in Turridae, Tateidae previously classified in Hydrobiidae, Truncatelloidea previously classified as Rissooidea.

<u>References cited in this table</u>

(a) Osca, D., Templado, J., & Zardoya, R. (2014). The mitochondrial genome of *Ifremeria nautilei* and the phylogenetic position of the enigmatic deep-sea Abyssochrysoidea (Mollusca: Gastropoda). *Gene*, *547*(2), 257-266.

(b) Guo, Y., Fu, Z., Feng, J., Ye, Y., Li, J., Guo, B., & Lv, Z. (2019). The complete mitochondrial genome and phylogenetic analysis of *Epitonium scalare* (Gastropoda, Epitoniidae). *Mitochondrial DNA Part B*, *4*(1), 1070-1071.

(c) Marques, J. P., Sotelo, G., Larsson, T., Johannesson, K., Panova, M., & Faria, R. (2017). Comparative mitogenomic analysis of three species of periwinkles: *Littorina fabalis*, *L. obtusata* and *L. saxatilis*. *Marine genomics*, *32*, 41-47.

(d) Fourdrilis, S., de Frias Martins, A. M., & Backeljau, T. (2018). Relation between mitochondrial DNA hyperdiversity, mutation rate and mitochondrial genome evolution in *Melarhaphe neritoides* (Gastropoda: Littorinidae) and other Caenogastropoda. *Scientific reports*, *8*(1), 1-12.

(e) Osca, D., Templado, J., & Zardoya, R. (2015). Caenogastropod mitogenomics. *Molecular Phylogenetics and Evolution*, *93*, 118-128.

(f) Li, P. Y., Yang, Y., Li, Y. G., & Sun, S. E. (2018). The complete mitochondrial genome of *Glossaulax reiniana* (Littorinimorpha: Naticidae). *Mitochondrial DNA Part B*, *3*(2), 1263-1264.

(g) Wang, Z., Liu, C., Liu, H., Wang, B., Pang, M., & Zheng, F. (2019). The mitochondrial genome of the marine gastropod *Neverita didyma* (Roding, 1798) (Mollusca: Gastropoda). *Mitochondrial DNA Part B*, *4*(1), 1545-1546.

(h) Rawlings, T. A., MacInnis, M. J., Bieler, R., Boore, J. L., & Collins, T. M. (2010). Sessile snails, dynamic genomes: gene rearrangements within the mitochondrial genome of a family of caenogastropod molluscs. *BMC genomics*, *11*(1), 440.

(i) Peretolchina, T. E., Sitnikova, T. Y., & Sherbakov, D. Y. (2018). The complete mitochondrial genomes of four endemic baikal mollusks (Mollusca: Caenogastropoda). *Механизмы регуляции функций органелл эукариотической клетки/Mame*, 87.

(j) Zhao, Q. P., Zhang, S. H., Deng, Z. R., Jiang, M. S., & Nie, P. (2010). Conservation and variation in mitochondrial genomes of gastropods *Oncomelania hupensis* and *Tricula hortensis*, intermediate host snails of Schistosoma in China. *Molecular phylogenetics and evolution*, *57*(1), 215-226.

(k) Neiman, M., Hehman, G., Miller, J. T., Logsdon Jr, J. M., & Taylor, D. R. (2010). Accelerated mutation accumulation in asexual lineages of a freshwater snail. *Molecular biology and evolution*, *27*(4), 954-963.

(l) Pu, L., Liu, H., Yang, M., Li, B., Xia, G., Shen, M., & Wang, G. (2019). Complete mitochondrial genome of tiger cowrie *Cypraea tigris* (Linnaeus, 1758). *Mitochondrial DNA Part B*, *4*(2), 2755-2756.

(m) Zhao, Z. Y., Tu, Z. G., Bai, L. R., & Cui, J. (2018). Characterization of an endangered marine strombid gastropod Strombus luhuanus complete mitochondrial genome. *Conservation genetics resources*, *10*(1), 55-57.

(n) Márquez, E. J., Castro, E. R., & Alzate, J. F. (2016). Mitochondrial genome of the endangered marine gastropod *Strombus gigas* Linnaeus, 1758 (Mollusca: Gastropoda). *Mitochondrial DNA Part A*, *27*(2), 1516-1517.

(o) Jiang, D., Zheng, X., Zeng, X., Kong, L., & Li, Q. (2019). The complete mitochondrial genome of *Harpago chiragra* and *Lambis lambis* (Gastropoda: Stromboidea): implications on the Littorinimorpha phylogeny. *Scientific reports*, *9*(1), 1-9.

(p) Cunha, R. L., Grande, C., & Zardoya, R. (2009). Neogastropod phylogenetic relationships based on entire mitochondrial genomes. *BMC Evolutionary Biology*, *9*(1), 210.

(q) Cho, I. Y., Kim, K. Y., Yi, C. H., Kim, I. H., Jung, Y. H., Hwang, S. J., ... & Kim, M. S. (2017). Full-length mitochondrial genome of the triton trumpet *Charonia lampas* (Littorinimorpha: Ranellidae). *Mitochondrial DNA Part B*, *2*(2), 759-760.

(r) Xu, M., Ye, Y., Yang, H., Xu, Z., Guo, B., Xu, K., ... & Li, P. (2019). The complete mitochondrial genome of *Onustus exutus* (Gastropoda: Xenophoridae). *Mitochondrial DNA Part B*, *4*(1), 989-990.

(s) Núñez-Acuña, G., Aguilar-Espinoza, A., & Gallardo-Escárate, C. (2013). Complete mitochondrial genome of *Concholepas concholepas* inferred by 454 pyrosequencing and mtDNA expression in two mollusc populations. *Comparative Biochemistry and Physiology Part D: Genomics and Proteomics*, *8*(1), 17-23.

(t) Sung, J. M., Karagozlu, M. Z., Lee, J., Kwak, W., & Kim, C. B. (2016). The complete mitochondrial genome of *Menathais tuberosa* (Gastropoda, Neogastropoda, Muricidae) collected from Chuuk Lagoon. *Mitochondrial DNA Part B*, *1*(1), 468-469.

(u) Sun, X., & Yang, A. (2016). The complete mitochondrial genome of *Rapana venosa* (Gastropoda, Muricidae). *Mitochondrial DNA Part A*, *27*(2), 1471-1472.

(v) Ki, J. S., Lee, Y. M., Jung, S. O., Horiguchi, T., Cho, H. S., & Lee, J. S. (2010). Mitochondrial genome of *Thais clavigera* (Mollusca: Gastropoda): Affirmation of the conserved, ancestral gene pattern within the mollusks. *Molecular phylogenetics and evolution*, *54*(3), 1016-1020.

(w) Zhong, S., Zhao, Y., Wang, X., Song, Z., & Zhang, Q. (2017). The complete mitochondrial genome of *Indothais lacera* (Neogastropoda: Muricidae). *Mitochondrial DNA Part B*, *2*(2), 877-878.

(x) Zhong, S., Huang, L., Huang, G., Liu, Y., & Wang, W. (2019). The first complete mitochondrial genome of *Murex* from *Murex trapa* (Neogastropoda: Muricidae). *Mitochondrial DNA Part B*, *4*(2), 3394-3395.

(y) Xiong, G., Ma, X., Wang, X. Q., Zhu, D. L., Wang, L. M., & Qin, Q. (2015). The complete mitochondrial genome of the *Babylonia lutosa*. *Mitochondrial DNA*, *26*(2), 187-188.

(z) Zhong, S., Huang, G., Liu, Y., & Huang, L. (2019). The complete mitochondrial genome of marine gastropod *Melo melo* (Neogastropoda: Volutoidea). *Mitochondrial DNA Part B*, *4*(2), 4161-4162.

(aa) Harasewych, M. G., Sei, M., Wirshing, H. H., González, V. L., & Uribe, J. E. (2019). The complete mitochondrial genome of *Neptuneopsis gilchristi* GB Sowerby III, 1898 (Neogastropoda: Volutidae: Calliotectinae). *NAUTILUS*, *133*(3-4), 67-73.

(bb) Xu, Y., Bao, X., Gao, L., He, C., Liu, W., & Chai, X. (2016). The complete mitochondrial genome of *Buccinum pemphigum* (Neogastropoda: Buccinidae). *Mitochondrial DNA Part B*, *1*(1), 98-99.

(cc) Hao, Z. L., Yang, L. M., Zhan, Y. Y., Tian, Y., Mao, J. X., Wang, L., & Chang, Y. Q. (2016). The complete mitochondrial genome of *Neptunea arthritica cumingii* Crosse (Gastropoda: Buccinidae). *Mitochondrial DNA Part B*, *1*(1), 220-221.

(dd) Bao, X., He, C., Su, H., Zhang, Z., Xu, Y., Li, Y., & Liu, W. (2016). Complete mitochondrial genome of *Volutharpa perryi* (Neogastropoda: Buccinidae). *Mitochondrial DNA Part B*, *1*(1), 88-89.

(ee) Vaux, F., Trewick, S. A., Crampton, J. S., Marshall, B. A., Beu, A. G., Hills, S. F., & Morgan-Richards, M. (2018). Evolutionary lineages of marine snails identified using molecular phylogenetics and geometric morphometric analysis of shells. *Molecular phylogenetics and evolution*, *127*, 626-637.

(ff) Jónsson, Z. O., Pálsson, S., Westfall, K. M., Magnúsdóttir, H., Goodall, J., & Ornolfsdottir, E. B. (2019). The mitochondrial genome of common whelk *Buccinum undatum* (Neogastropoda: Buccinidae). *Mitochondrial DNA Part B*, *4*(1), 457-459.

(gg) Tian, Y., Hao, Z., Zhu, J., Yang, J., Hao, S., & Chang, Y. (2019). The complete mitochondrial genome of *Lirabuccinum musculu* Collomon Et Lawless, 2013. *Mitochondrial DNA Part B*, *4*(1), 1185-1186.

(hh) Simison, W. B., Lindberg, D. R., & Boore, J. L. (2006). Rolling circle amplification of metazoan mitochondrial genomes. *Molecular phylogenetics and evolution*, *39*(2), 562-567.

(ii) Yang, H., Ye, Y., Liu, S., Xu, M., & Guo, B. (2019). Characterization of complete mitochondrial genome of *Nassarius hepaticus* (Stenoglossa, Nassariidae). *Mitochondrial DNA Part B*, *4*(1), 446-447.

(jj) Yang, Y., Li, Q., Kong, L., & Yu, H. (2019). Mitogenomic phylogeny of *Nassarius* (Gastropoda: Neogastropoda). *Zoologica Scripta*, *48*(3), 302-312.

(kk) Yang, Y., Li, Q., Kong, L., & Yu, H. (2018). Comparative mitogenomic analysis reveals cryptic species in *Reticunassa festiva* (Neogastropoda: Nassariidae). *Gene*, *662*, 88-96.

(ll) Zhong, S., Huang, L., Huang, G., Liu, Y., & Xu, W. (2019). The first complete mitochondrial genome of Melongenidae from *Hemifusus tuba* (Neogastropoda: Buccinoidea). *Mitochondrial DNA Part B*, *4*(2), 3400-3401.

(mm) Uribe, J. E., Puillandre, N., & Zardoya, R. (2017). Beyond Conus: phylogenetic relationships of Conidae based on complete mitochondrial genomes. *Molecular phylogenetics and evolution*, *107*, 142-151.

(nn) Chen, P. W., Hsiao, S. T., Chen, K. S., Tseng, C. T., Wu, W. L., & Hwang, D. F. (2016). The complete mitochondrial genome of *Conus capitaneus* (Neogastropoda: Conidae). *Mitochondrial DNA Part B*, *1*(1), 520-521.

(oo) Chen, P. W., Hsiao, S. T., Huang, C. W., Chen, K. S., Tseng, C. T., Wu, W. L., & Hwang, D. F. (2016). The complete mitochondrial genome of *Conus tulipa* (Neogastropoda: Conidae). *Mitochondrial DNA Part A*, *27*(4), 2738-2739.

(pp) Abalde, S., Tenorio, M. J., Afonso, C. M., Uribe, J. E., Echeverry, A. M., & Zardoya, R. (2017). Phylogenetic relationships of cone snails endemic to Cabo Verde based on mitochondrial genomes. *BMC evolutionary biology*, *17*(1), 231.

(qq) Chen, P. W., Wu, W. L., & Hwang, D. F. (2018). The complete mitochondrial genome of *Conus quercinus* (Neogastropoda: Conidae). *Mitochondrial DNA Part B*, *3*(2), 933-934.

(rr) Uribe, J. E., Zardoya, R., & Puillandre, N. (2018). Phylogenetic relationships of the conoidean snails (Gastropoda: Caenogastropoda) based on mitochondrial genomes. *Molecular phylogenetics and evolution*, *127*, 898-906.

(ss) Fu, Z., Feng, J., Guo, Y., Ye, Y., Li, J., Guo, B., & Lv, Z. (2019). The complete mitochondrial genome and phylogenetic analysis of *Turricula nelliae spurius* (Gastropoda, Turridae). *Mitochondrial DNA Part B*, *4*(1), 1068-1069.

(tt) Bandyopadhyay, P. K., Stevenson, B. J., Cady, M. T., Olivera, B. M., & Wolstenholme, D. R. (2006). Complete mitochondrial DNA sequence of a Conoidean gastropod, *Lophiotoma* (Xenuroturris) *cerithiformis*: gene order and gastropod phylogeny. *Toxicon*, *48*(1), 29-43.

(uu) McComish, B. J., Hills, S. F., Biggs, P. J., & Penny, D. (2010). Index-free de novo assembly and deconvolution of mixed mitochondrial genomes. *Genome biology and evolution*, *2*, 410-424.

(vv) Wang, P., Zhu, P., Wu, H., Xu, Y., Liao, Y., & Zhang, H. (2019). The complete mitochondrial genome of *Neritina violacea*. *Mitochondrial DNA Part B*, *4*(2), 2942-2943.

(ww) Cho, I. Y., Kim, K. Y., Jung, T. W., Yi, C. H., Kim, I. H., Hong, S. S., ... & Kim, M. S. (2017). Complete sequence analysis of mitochondrial genome of *Clithon retropictum* (Gastropoda; Neritidae). *The Korean Journal of Malacology*, *33*(3), 165-171.

(xx) Kurabayashi, A., & Ueshima, R. (2000). Complete sequence of the mitochondrial DNA of the primitive opisthobranch gastropod *Pupa strigosa*: systematic implication of the genome organization. *Molecular Biology and Evolution*, *17*(2), 266-277.

(yy) Knudsen, B., Kohn, A. B., Nahir, B., McFadden, C. S., & Moroz, L. L. (2006). Complete DNA sequence of the mitochondrial genome of the sea-slug, *Aplysia californica*: conservation of the gene order in Euthyneura. *Molecular phylogenetics and evolution*, *38*(2), 459-469.

(zz) Grande, C., Templado, J., Cervera, J. L., & Zardoya, R. (2004). Phylogenetic relationships among Opisthobranchia (Mollusca: Gastropoda) based on mitochondrial cox 1, trnV, and rrnL genes. *Molecular phylogenetics and Evolution*, *33*(2), 378-388.

(aaa) Irwin, A. R., Strong, E. E., Kano, Y., Harper, E. M., & Williams, S. T. (2021). Eight new mitogenomes clarify the phylogenetic relationships of Stromboidea within the caenogastropod phylogenetic framework. *Molecular Phylogenetics and Evolution*, *158*, 107081.

(bbb) Lee, H. T., Liao, C. H., Huang, C. W., Chang, Y. C., & Hsu, T. H. (2021). The complete mitochondrial genome of *Laevistrombus canarium* (Gastropoda: Stromboidae). *Mitochondrial DNA Part B*, *6*(2), 591-592.

(ccc) Zhong, S., Liu, Y., Huang, G., & Huang, L. (2020). The first complete mitochondrial genome of Bursidae from *Bufonaria rana* (Caenogastropoda: Tonnoidea). *Mitochondrial DNA Part B*, *5*(3), 2585-2586.

(ddd) Sanders, M. T., Merle, D., Laurin, M., Bonillo, C., & Puillandre, N. (2021). Raising names from the dead: a time-calibrated phylogeny of frog shells (Bursidae, Tonnoidea, Gastropoda) using mitogenomic data. *Molecular Phylogenetics and Evolution*, *156*, 107040.

(eee) Wang, Q., Liu, H., Yue, C., Xie, X., Li, D., Liang, M., & Li, Q. (2021). Characterization of the complete mitochondrial genome of *Ficus variegata* (Littorinimorpha: Ficidae) and molecular phylogeny of Caenogastropoda. *Mitochondrial DNA Part B*, *6*(3), 1126-1128.

(fff) Fukumori, H., Itoh, H., & Irie, T. (2019). The mitochondrial genome of the gold-ringed cowry *Monetaria annulus* (Mollusca: Gastropoda: Cypraeidae) determined by whole-genome sequencing. *Mitochondrial DNA Part B*, *4*(2), 2305-2307.

(ggg) Uribe, J. E., Fedosov, A. E., Murphy, K. R., Sei, M., & Harasewych, M. G. (2021). The complete mitochondrial genome of *Costapex baldwinae* (Gastropoda: Neogastropoda: Turbinelloidea: Costellariidae) from the Caribbean Deep-Sea. *Mitochondrial DNA Part B*, *6*(3), 943-945.

(hhh) Feng, J., Fu, Z., Guo, Y., Ye, Y., Li, J., Guo, B., & Lü, Z. (2019). The complete mitochondrial genome and phylogenetic analysis of *Fusinus longicaudus* (Gastropoda: Fasciolariidae). *Mitochondrial DNA Part B*, *4*(1), 1943-1944.
