# Supplementary material for: The queen conch mitogenome: intra- and interspecific mitogenomic variability in Strombidae and phylogenetic considerations within the Hypsogastropoda
Source: Sci Rep. 2021 Jun 7;11:11972. doi: 10.1038/s41598-021-91224-0 (PMC8184947; doi:10.1038/s41598-021-91224-0)
Supplement: Supplementary file 2 — Supplementary Information 2. [file 41598_2021_91224_MOESM2_ESM.pdf]

The queen conch mitogenome: Intra- and interspecific mitogenomic variability in Strombidae and phylogenetic considerations within the Hypsogastropoda

Salima Machkour-M'Rabet, Margaret M. Hanes, Josué Jacob Martínez-Noguez, Jorge Cruz-Medina, Francisco J. García-De León

**Supplementary Figure S1**. Relative synonymous codon usage (RSCU) for the nine mitogenomes of Strombidae belonging to eight species. The 20 amino acid are designated using the three and one abbreviations, and codon stop is also presented. Species are: *Conomurex luhuanus*, *Harpago chiragra*, *Lambis lambis*, *Aliger gigas*, *Aliger gigas** (this ultimate from this study), *Laevistrombus canarium*, *Ministrombus variabilis*, *Strombus pugilis*, and *Tridentarius dentatus*.

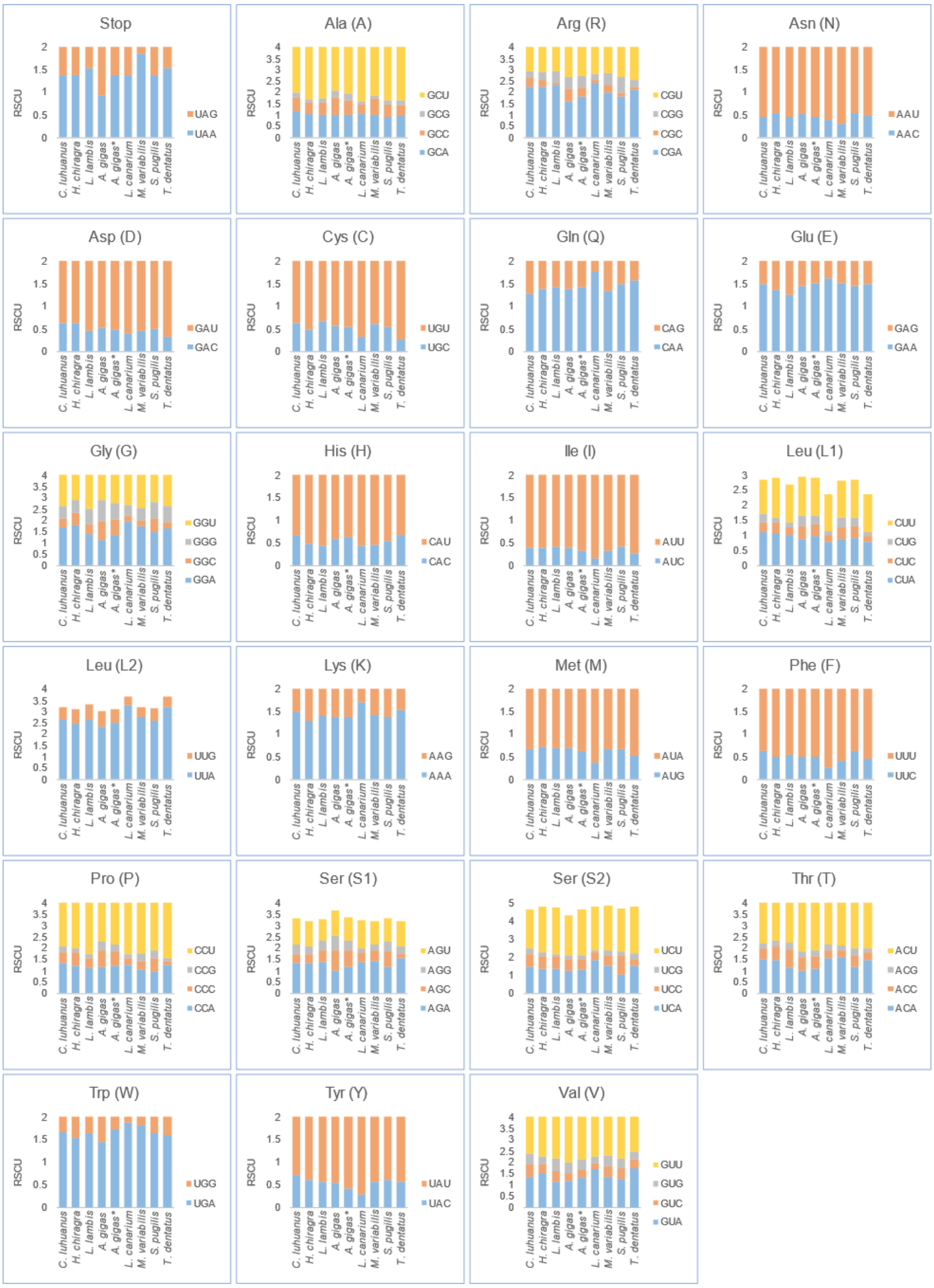

**Supplementary Table S2**. Nucleotide composition for *Aliger gigas* and other Strombidae species available in GenBank. (#) *A. gigas* from this study (MZ157283), *A. gigas* from the accession number NC024932, *C. luhuanus* (KY853669), *H. chiragra* (MH122656), *L. lambis* (MH115428), *L. canarium* (MT937083), *M. variabilis* (MW244824), *S. pugilis* (MW244819), and *T. dentatus* (MW244820), * values based on incomplete genome (only 18 tRNAs).

| | Species | Whole genome | 13 PCGs | 2 rRNAs | 22 tRNAs |
|---|---|---|---|---|---|
| AT content (%) | *Aliger gigas* (#) | 65.8 | 65.3 | 67.1 | 65.3 |
| | *Aliger gigas* | 65.8 | 65.3 | 67.1 | 65.2 |
| | *Conomurex luhuanus* | 65.7 | 65.2 | 67.4 | 64.8 |
| | *Harpago chiragra* | 66.2 | 65.7 | 67.3 | 65.9 |
| | *Lambis lambis* | 66.1 | 65.7 | 67.0 | 65.3 |
| | *Laevistrombus canarium* | 69.9 | 69.6 | 70.4 | 67.8 |
| | *Ministrombus variabilis* | 66.9* | 66.3 | 68.3 | 66.8* |
| | *Strombus pugilis* | 65.3 | 64.7 | 66.5 | 65.5 |
| | *Tridentarius dentatus* | 68.2 | 67.8 | 69.5 | 66.2 |
| AT skew | *Aliger gigas (#)* | -0.13 | -0.19 | 0.07 | 0.03 |
| | *Aliger gigas* | -0.13 | -0.19 | 0.08 | 0.03 |
| | *Conomurex luhuanus* | -0.10 | -0.16 | 0.08 | 0.02 |
| | *Harpago chiragra* | -0.13 | -0.19 | 0.06 | 0.02 |
| | *Lambis lambis* | -0.13 | -0.20 | 0.08 | 0.03 |
| | *Laevistrombus canarium* | -0.11 | -0.17 | 0.06 | 0.04 |
| | *Ministrombus variabilis* | -0.13* | -0.18 | 0.06 | 0.04* |
| | *Strombus pugilis* | -0.13 | -0.20 | 0.08 | 0.04 |
| | *Tridentarius dentatus* | -0.12 | -0.18 | 0.06 | 0.05 |
| GC skew | *Aliger gigas #* | 0.03 | 0.01 | 0.15 | 0.14 |
| | *Aliger gigas* | 0.03 | < 0.01 | 0.15 | 0.14 |
| | *Conomurex luhuanus* | < 0.01 | -0.01 | 0.10 | 0.14 |
| | *Harpago chiragra* | 0.02 | -0.01 | 0.13 | 0.16 |
| | *Lambis lambis* | 0.03 | 0.01 | 0.12 | 0.14 |
| | *Laevistrombus canarium* | 0.03 | 0.01 | 0.13 | 0.16 |
| | *Ministrombus variabilis* | 0.11* | < 0.01 | 0.15 | 0.17* |
| | *Strombus pugilis* | 0.02 | -0.01 | 0.16 | 0.15 |
| | *Tridentarius dentatus* | 0.04 | 0.03 | 0.14 | 0.13 |

**Supplementary Table S3**. Start and Stop codon used among Strombidae species. (#) *A. gigas* from this study, (*) to identify the difference between information from publication and from the mitogenome, red color to highlight a different codon from the common ATG start codon or TAA stop codon. Letters refer to the publications: (a) Zhao et al. 2018, (b) Jiang et al. 2019, (c) Irwin et al. 2021, (d) Lee et al. 2021.

| | **Strombidae species available in GenBank** | | | | | | | |
|---|---|---|---|---|---|---|---|---|
| | ***A. gigas#*** | ***C. luhuanus***[a] | ***H. chiragra***[b] | ***L. lambis***[b] | ***S. pugilis***[c] | ***M. variabilis***[c] | ***T. dentatus***[c] | ***L. canarium***[d] |
| **PCGs** | **Start codon** | | | | | | | |
| COX1 | ATG | ATG | ATG | ATG | ATG | ATG | ATG | ATG |
| COX2 | ATG | ATG | ATG | ATG | ATG | ATG | ATG | ATG |
| ATP8 | ATG | ATG | ATG | ATG | ATG | ATG | ATG | ATG |
| ATP6 | ATG | ATA* | ATG | ATG | ATG | ATG | ATG | ATG |
| NAD1 | ATG | ATG | ATG | ATG | ATG | ATG | ATG | ATG |
| NAD6 | ATG | ATG | ATG | ATG | ATG | ATG | ATG | ATG |
| CytB | ATG | ATT* | ATG | ATG | ATG | ATG | ATG | ATG |
| NAD4L | ATG | ATT* | ATG | ATG | ATG | ATG | ATG | ATG |
| NAD4 | GTG | ATT* | GTG | ATG | ATG* | ATG* | ATG* | ATG |
| NAD5 | ATG | ATG* | ATG | ATG | ATG | ATG | ATG | ATG |
| COX3 | ATG | ATG | ATG | ATG | ATG | ATG | ATG | ATG |
| NAD3 | ATG | ATG* | ATG | ATG | ATG | ATG | ATG | ATG |
| NAD2 | ATG | ATA | ATG* | ATG* | ATG | ATG | ATG | ATG |
| **PCGs** | **Stop codon** | | | | | | | |
| COX1 | TAA | TAA | TAG | TAA | TAA | TAA | TAA | TAA |
| COX2 | TAA | TAA | TAA | TAA | TAA | TAA | TAA | TAA |
| ATP8 | TAA | TAA | TAA | TAA | TAA | TAA | TAA | TAA |
| ATP6 | TAA | TAA | TAA | TAA | TAA | TAA | TAG | TAG |
| NAD1 | TAG | TAG* | TAG | TAG | TAA | TAA | TAA | TAG |
| NAD6 | TAG | TAA | TAA | TAA | TAA | TAA | TAA | TAA |
| CytB | TAA | TAA* | TAA | TAA | TAA | TAA | TAA | TAA |
| NAD4L | TAG | TAG* | TAG | TAG | TAG | TAG | TAG | TAG |
| NAD4 | TAA | TAA* | TAA | TAA | TAG | TAA | TAA* | TAA |
| NAD5 | TAA | TAG* | TAA | TAA | TAA | TAA | TAG | TAA |
| COX3 | TAA | TAG* | TAA | TAA | TAG | TAA | TAA | TAA |
| NAD3 | TAG | TAA* | TAA | TAA | TAA | TAA | TAA | TAG |
| NAD2 | TAA | TAA* | TAG | TAG | TAG | TAA | TAA | TAA |

**Supplementary Table S4**. Intra- and interspecific statistics of mitogenomes variation in eight Strombidae species (*Conomurex luhuanus*, *Harpago chiragra*, *Lambis lambis*, *Aliger gigas*, *Ministrombus variabilis*, *Strombus pugilis*, *Laevistrombus canarium*, *Tridentarius dentatus*). SNP: total number of single nucleotide polymorphism, InDel: total number of indel (insertion and deletion) sites with the total number of indel event inside bracket, Syn Subs.: total number of synonymous substitutions, Nonsyn Subs.: total number of nonsynonymous substitutions with the percentage of change in AA in the gene inside brackets, π: nucleotide diversity.

| | Intraspecific variation | | | | | Interspecific variation | | | | |
|---|---|---|---|---|---|---|---|---|---|---|
| | SNP | InDel | Syn Subs. | Nonsyn Subs. | π | SNP | InDel | Syn Subs. | Nonsyn Subs. | π |
| Whole genome | 115 | 5 (4) | 76 | 15 | 0.0074 | 5228 | 1002 (294) | 4674 | 1022 | 0.1698 |
| 13 PCGs | 94 | 25 (2) | 76 | 15 | 0.0084 | 4181 | 138 (5) | 4674 | 1036 | 0.1778 |
| COX1 | 10 | 0 | 8 | 1 (0.2%) | 0.0065 | 476 | 0 | 616 | 4 (0.7%) | 0.1416 |
| COX2 | 4 | 0 | 4 | 0 (0.0%) | 0.0058 | 200 | 0 | 224 | 14 (6.1%) | 0.1370 |
| ATP8 | 3 | 0 | 2 | 1 (1.9%) | 0.0189 | 59 | 0 | 41 | 29 (55.8%) | 0.1653 |
| ATP6 | 4 | 0 | 4 | 0 (0.0%) | 0.0057 | 230 | 48 (1) | 316 | 21 (9.8%) | 0.1722 |
| NAD1 | 5 | 0 | 4 | 1 (0.3%) | 0.0053 | 338 | 0 | 397 | 49 (15.8%) | 0.1635 |
| NAD6 | 5 | 0 | 5 | 0 (0.0%) | 0.0099 | 229 | 3 (1) | 206 | 102 (61.1%) | 0.2185 |
| CytB | 12 | 0 | 8 | 3 (0.8%) | 0.0105 | 384 | 12 (1) | 474 | 51 (13.6%) | 0.1616 |
| NAD4L | 1 | 0 | 1 | 0 (0.0%) | 0.0034 | 102 | 24 (1) | 141 | 8 (8.9%) | 0.1741 |
| NAD4 | 15 | 0 | 14 | 1 (0.2%) | 0.0109 | 570 | 30 (1) | 573 | 224 (50.2%) | 0.2016 |
| NAD5 | 14 | 25 (2) | 9 | 5 (0.9%) | 0.0081 | 730 | 0 | 774 | 229 (39.9%) | 0.2053 |
| COX3 | 9 | 0 | 8 | 1 (0.4%) | 0.0115 | 249 | 0 | 317 | 28 (10.8%) | 0.1529 |
| NAD3 | 2 | 0 | 1 | 0 (0.0%) | 0.0057 | 130 | 0 | 133 | 30 (25.6%) | 0.1633 |
| NAD2 | 10 | 0 | 7 | 3 (0.9%) | 0.0094 | 473 | 27 (1) | 450 | 220 (64.1%) | 0.2214 |
